# Implicit Counterfactual Effect in Partial Feedback Reinforcement Learning: Behavioral and Modeling Approach

**DOI:** 10.3389/fnins.2022.631347

**Published:** 2022-05-10

**Authors:** Zahra Barakchian, Abdol-Hossein Vahabie, Majid Nili Ahmadabadi

**Affiliations:** ^1^Department of Cognitive Neuroscience, Institute for Research in Fundamental Sciences, Tehran, Iran; ^2^Cognitive Systems Laboratory, Control and Intelligent Processing Center of Excellence, School of Electrical and Computer Engineering, College of Engineering, University of Tehran, Tehran, Iran; ^3^Department of Psychology, Faculty of Psychology and Education, University of Tehran, Tehran, Iran

**Keywords:** reinforcement learning, value learning, contextual effect, counterfactual outcome, partial and complete feedback

## Abstract

Context remarkably affects learning behavior by adjusting option values according to the distribution of available options. Displaying counterfactual outcomes, the outcomes of the unchosen option alongside the chosen one (i.e., providing complete feedback), would increase the contextual effect by inducing participants to compare the two outcomes during learning. However, when the context only consists of the juxtaposition of several options and there is no such explicit counterfactual factor (i.e., only partial feedback is provided), it is not clear whether and how the contextual effect emerges. In this research, we employ Partial and Complete feedback paradigms in which options are associated with different reward distributions. Our modeling analysis shows that the model that uses the outcome of the chosen option for updating the values of both chosen and unchosen options in opposing directions can better account for the behavioral data. This is also in line with the diffusive effect of dopamine on the striatum. Furthermore, our data show that the contextual effect is not limited to probabilistic rewards, but also extends to magnitude rewards. These results suggest that by extending the counterfactual concept to include the effect of the chosen outcome on the unchosen option, we can better explain why there is a contextual effect in situations in which there is no extra information about the unchosen outcome.

## 1. Introduction

Behavior necessarily occurs within a specific context. A wealth of studies have investigated the effect of context on decision making (Summerfield and Tsetsos, 2015; Rigoli et al., 2016a,b, 2017, 2018; Tsetsos et al., 2016), while the effect of context on reinforcement learning has received little attention. Recent studies have shown that many cognitive biases arise due to the effect of the context in which the value learning process occurs (Palminteri et al., 2015; Klein et al., 2017; Bavard et al., 2018). The choice context is comprised of the currently available options. Two paradigms have been implemented to investigate the value learning process. In the Complete feedback paradigm, participants are shown the outcomes of the options they select (factual outcomes) as well as the outcomes of the options they forgo (counterfactual outcomes). Thus, participants are able to compare the factual and counterfactual outcomes and thereby learn the value of the selected option relative to the value of the forgone option (Palminteri et al., 2015; Bavard et al., 2018). In the Partial feedback paradigm, participants are only shown the outcomes of the selected options, so they are not able to compare the two outcomes. It is unknown if and how the contextual effect appears in the Partial feedback paradigm.

In reinforcement learning, the value of an option is usually learned through trial and error (Sutton and Barto, 2018). Reinforcement learning is an incremental process in which option values are updated *via* prediction errors, that is, the difference between the received reward versus the expected reward (Sutton and Barto, 2018). Prediction errors are encoded in the brain by the neurotransmitter dopamine (Schultz et al., 1997). Dopamine releases diffusively and has opposing excitatory and inhibitory effects on two distinct populations of striatal neurons called D1-SPNs and D2-SPNs (spiny projection neurons), respectively. These two clusters encode the values of the two competing options (Frank et al., 2004; Tai et al., 2012; Collins and Frank, 2014; Donahue et al., 2018; Nonomura et al., 2018; Shin et al., 2018; Bariselli et al., 2019). Inspired by the opposing effects of dopamine on D1- and D2-SPNs, we propose a simple reinforcement learning model called the Opposing Learning (OL) model. In the OL model, the chosen prediction error not only updates the value of the chosen option, but also that of the unchosen option, in opposite directions. Moreover, the updating of both option values depends on the observed rewards of the chosen option as well as those of the unchosen option. This implies that two competing options with identical expected rewards will have different learned values in different contexts.

In a typical value learning task, participants aim to maximize expected rewards. However, in the Complete feedback paradigm, in which counterfactual outcomes are also presented, the value learning strategy can be more complex (Palminteri et al., 2015; Klein et al., 2017; Bavard et al., 2018): Participants aim to learn option values by comparing the two outcomes relative to each other. This comparison will trigger regret (when the factual outcome is the less favorable) or relief [when the counterfactual outcome is the less favorable]. In an attempt to minimize regret and maximize relief, people aim to optimize the outcome difference, i.e., [*outcome*_*factual*_ − *outcome*_*counterfactual*_] (Camille et al., 2004; Coricelli et al., 2005, 2007). Recent studies have shown that people are neither fully expected-reward optimizers nor fully outcome-difference optimizers; they are hybrid optimizers who use both of these strategies but weight them differently (Kishida et al., 2016; Bavard et al., 2018). The individual differences between people depend on the degree to which a person utilizes each of these strategies. By adding a hybrid component to the simple OL model, we extend the OL model to account for the results in the Complete feedback paradigm as well.

Most of the previous studies have aimed to explain the contextual effects as resulting from the effect of the forgone outcome on the chosen value. In this study, we go beyond that explanation and aim to explain the contextual effect as resulting from the effect of the factual outcome on the unchosen value, especially in situations in which there is no forgone outcome. To this end, we designed two types of feedback paradigms, with and without forgone outcomes, and will show that we observed the contextual effect in both feedback paradigms. We introduce a novel reinforcement learning model that is better able to account for the underlying contextual bias in behavioral data than previous models. To study situations that occur frequently in everyday life, we use reward magnitude rather than reward probability and thereby show that the contextual effect is also present in paradigms using reward magnitude.

## 2. Results

### 2.1. Behavioral Task

**Figure 1:**
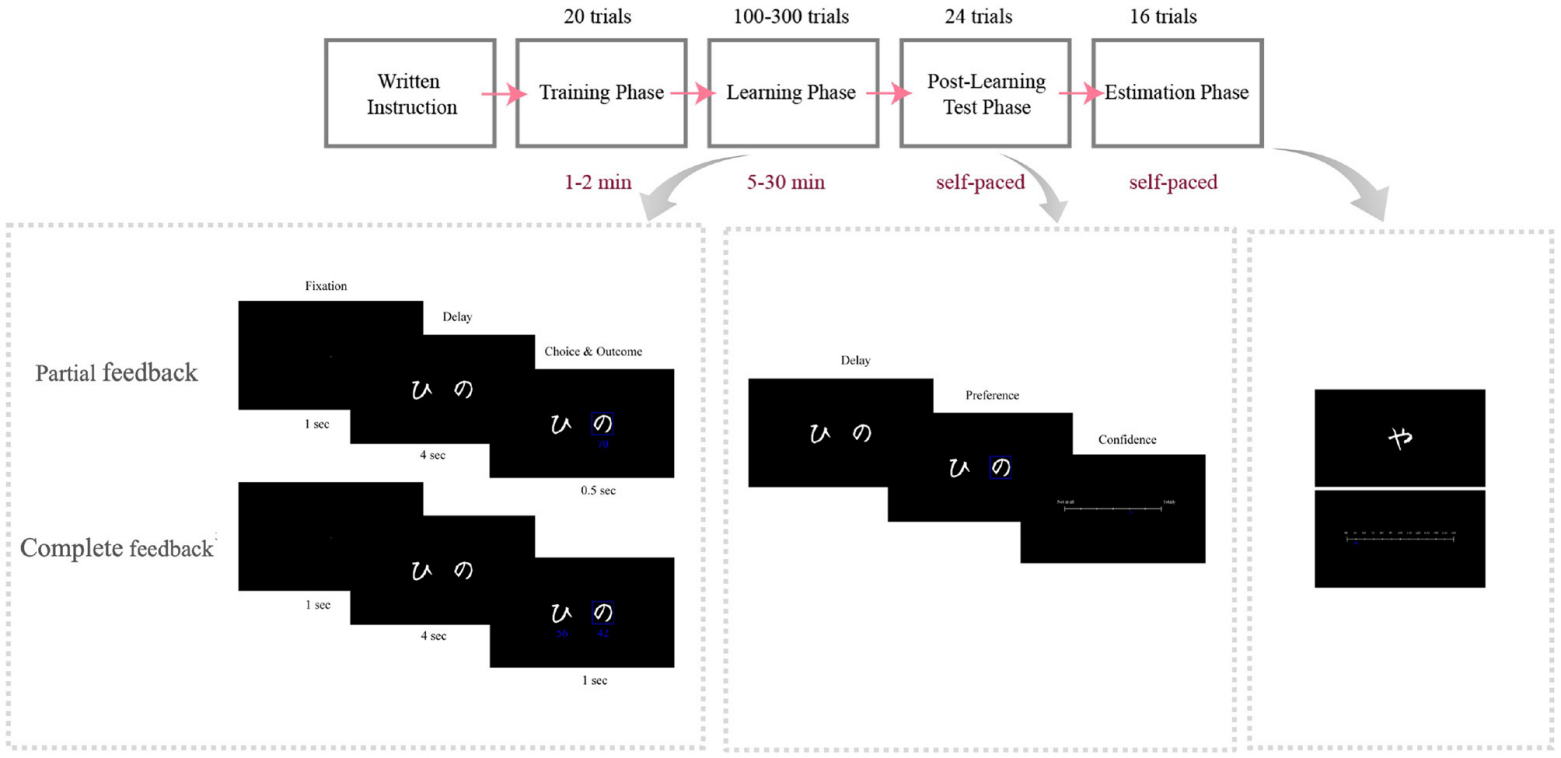
Behavioral design. Timelines of the Partial and Complete feedback versions of the task. Participants were given written instructions and trained through 20 trials before beginning the main task. They learned two pairs of options in the Learning phase by trial and error. In the subsequent transfer phase, they were presented with two options and were to choose the more advantageous option and then report their level of confidence about their choice. In the transfer phase, all possible binary combinations of options were presented. Finally, in the value estimation phase, they were to estimate the option value on a scale from 0 to 100.

Two groups of participants performed two different versions of the instrumental learning task: the Partial feedback version, in which we only provided them with factual outcomes, and the Complete feedback version, in which we provided them with both factual and counterfactual outcomes. Participants were to gain the most possible rewards during the task. The rewards were random independent numbers drawn from specified normal distributions. Participants faced two pairs of options (*A*_1_, *B*) and (*A*_2_, *C*), where *A*_1_ and *A*_2_ were associated with rewards from the same distribution as N(64, 13) and *B* and *C* were associated with rewards from two different distributions N(54, 13) and N(44, 13), respectively. To conceal the task structure from the participants, different images were assigned to *A*_1_ and *A*_2_, although their associated values were equal. After the learning phase, the participants unexpectedly entered the post-learning transfer phase in which all possible binary combinations of options (six pairs) were presented to them (each combination presented four times), and they were asked to choose the option with the highest expected reward. The transfer phase design aims to reveal any bias between *A*_2_ and *A*_1_. Similar designs can be found in the context-dependent value learning literature (Palminteri et al., 2015; Klein et al., 2017; Bavard et al., 2018). To avoid interfering with the participants' previous learning, no feedback was provided in the transfer phase (Frank et al., 2004, 2007; Palminteri et al., 2015; Klein et al., 2017; Bavard et al., 2018). After each choice, participants reported their confidence in that choice on a scale of 0 to 100. Finally, in the value estimation phase, participants reported their estimated expected value of each stimulus on a scale of 0–100 (Figure 1).

### 2.2. Performance

First, to see whether the participants had learned the option values during the task, we assessed their performance in the learning phase by calculating the percentage of trials in which they chose the advantageous option (the option with the higher expected reward). We observed that, in both versions of the task, the participants' performance was significantly better than random (0.5) [Partial: performance = 0.7613±0.1130; *t*-test, *p* = 1.1041*e* − 15, *t*_(34)_ = 13.6787, Complete: performance = 0.8823±0.0853, *t*-test, *p* = 2.8382*e* − 29, *t*_(41)_ = 29.0489; **Figure 3A**]. We also compared the participants' performance in the two versions of the task and found that their performance was significantly better in the complete feedback version [*p* = 4.5603e − 07, *t*_(75)_ = 5.3522, one-tailed *t*-test]. This means that providing information about counterfactual outcomes to participants facilitated their learning. This result is consistent with the previous studies (Palminteri et al., 2015; Klein et al., 2017; Bavard et al., 2018).

We also observed that participants' performance was significantly better than random (0.5) in the transfer phase [Partial: performance = 0.8786±0.2868, *t*-test, *p* = 8.7844*e* − 22, *t*_(34)_ = 21.673; Complete: performance = 0.9226±0.2618, *t*-test, *p* = 2.4064*e* − 24, *t*_(41)_ = 21.6362; Supplementary Figure 1]. Additionally, the reported confidence was significantly higher when participants had chosen the advantageous option than when they had chosen the non-advantageous option (Partial: average confidences of advantageous options = 0.7533±0.1895, average confidences of non-advantageous options = 0.4882±0.2326; Complete: average confidences of advantageous options = 0.7961±0.1818, average confidences of non-advantageous options = 0.5752±0.2124).

To determine whether the two versions of the task had different reward sensitivities, we ran a hierarchical model as follows. *action* ~ 1 + *vdif* * *task* + (1 + *vdif* * *task*|*subject*), where the *action* variable represents choosing the left option, the *vdif* variable is the option values difference, *task* variable is a categorical variable with 1 for the Partial and 2 for the Complete feedback version, and *subject* is the random effect variable. As can be seen in Supplementary Table 1, reward sensitivity was significantly higher in the Complete feedback version than in the Partial feedback version (*p*-value of the *vdif*:*task*2 regressor is 8.0551*e* − 17). For these and the following analyses, unanswered trials in the learning phase were excluded.

### 2.3. Contextual Effect

**Figure 2:**
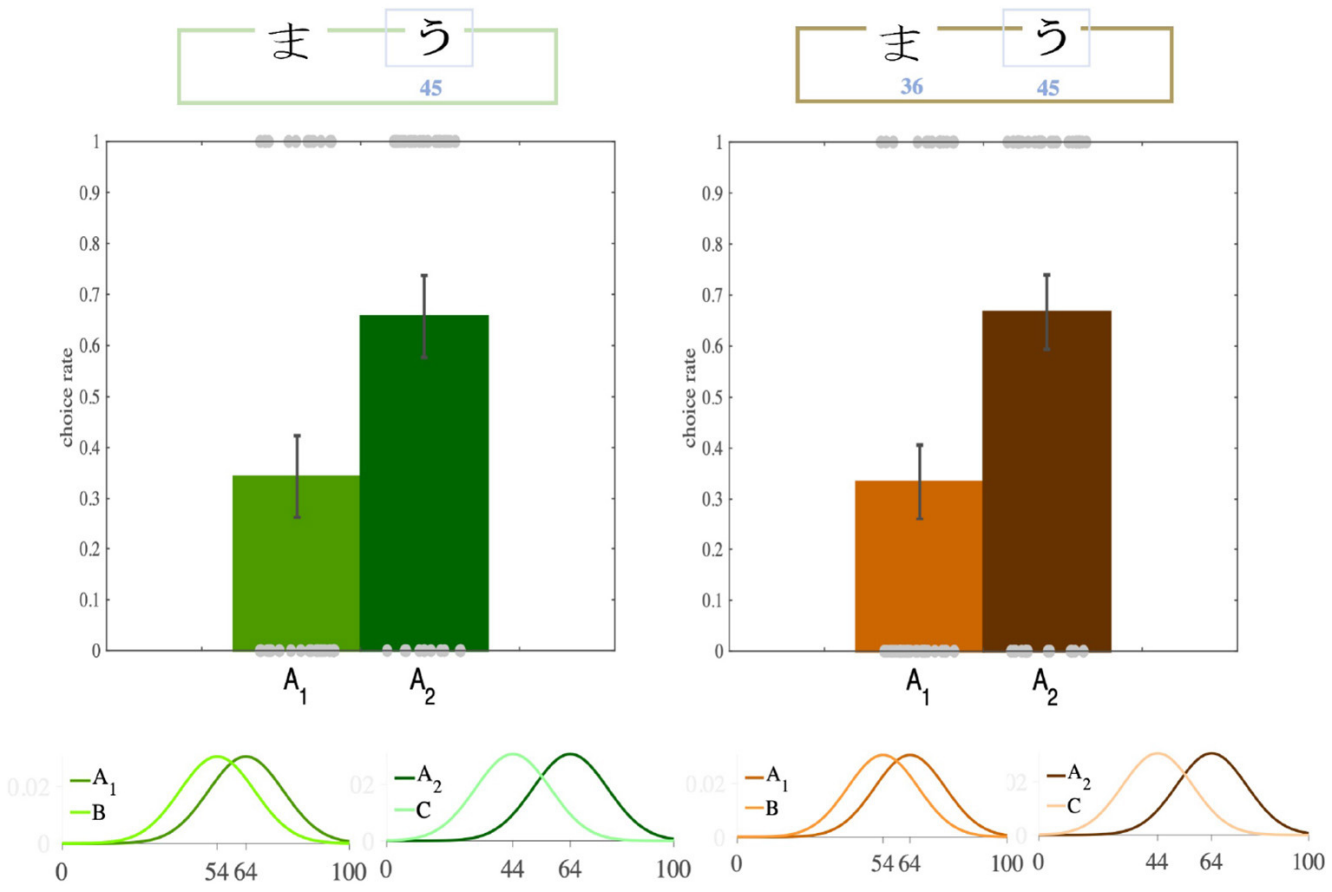
Transfer effect. In the transfer phase of the Partial and the Complete feedback versions of the task, participants significantly more often preferred the option with higher relative value (*A*_2_, dark colors), although the both options had equal absolute value.

**Figure 3:**
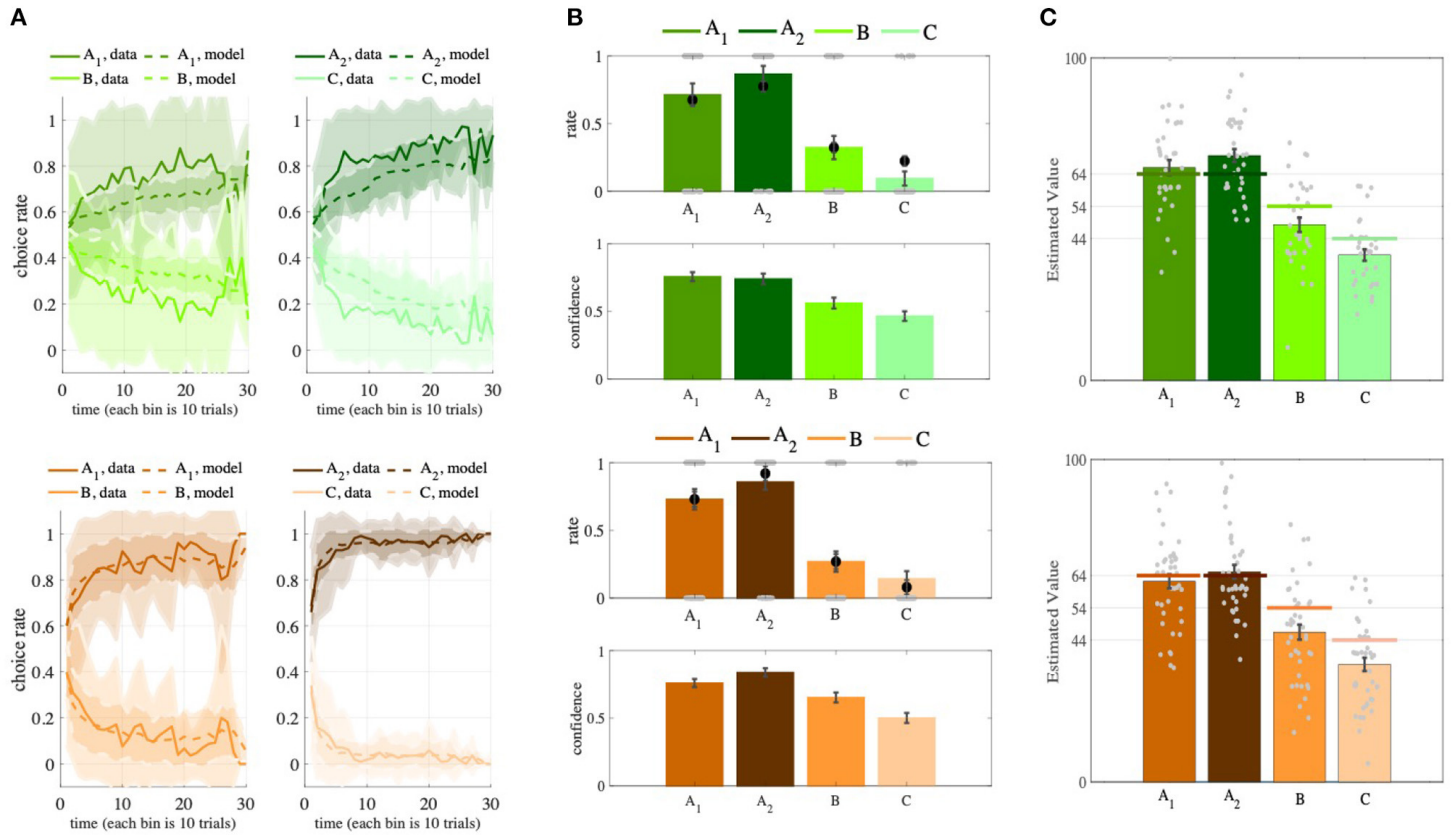
Behavioral results in the learning, transfer, and estimation phases. **(A)** The learning curves show that, when presented with paired options, participants learned to choose the more advantageous option in the pair (A*A*_1_ in *A*_1_*B* pair and *A*_2_ in *A*_2_*C* pair). The learning curve of the OL models shows similar results. Each bin in the x-axis is the average of choices in 10 trials. Solid lines show the behavioral data, dashed lines show the synthetic data. **(B)** The summarized preferences of the participants in six combinations (top) and their corresponding confidence levels (bottom), along with the predictions of the OL model (black dots). **(C)** The participants' value estimations (colored bars) are very close to the real expected rewards of the options *A*_1_ and *A*_2_ (colored lines). The Partial version is green and the Complete version is brown. Shadings denote *SD* and error bars denote *SEM*.

After the participants had learned the option values, we turned to the transfer phase to see whether there was any contextual effect. We found that participants' preferences between *A*_1_ and *A*_2_ had been significantly modulated by their distance from their paired options, such that despite having equal absolute values, participants preferred *A*_2_ over *A*_1_ (*transfer bias*) in both versions (Partial: *p* = 0.04, *ratio* = 0.65; Complete: *p* = 0.01, *ratio* = 0.66; binomial test; Figures 2, 3B, Supplementary Figure 1). Although this analysis has bee done on the first iterations of the participants choices in the transfer phase, this trend still remained after we considered all four iterations of *A*_1_ and *A*_2_ (the rates of choosing *A*_2_ over *A*_1_ for each participant), though it lost significance (Partial: *p* = 0.083; Complete: *p* = 0.063; *t*-test). This loss of significance might be explained as follows. In the learning phase, only certain pairs of options appeared together, allowing participants to compare and learn the options' relative values. However, in the transfer phase, the participants were presented with pairs of options that had not previously been paired so they were not able to compare the options' relative values. It may thus have been a better strategy not to rely completely on the relative values, but to use the absolute values of the options (For details of the binomial test see Supplementary Material).

To ensure that the observed bias in the transfer phase was a result of context-dependent value learning, and not of confounding factors, we examined which other factors could have affected the participants' preference for *A*_2_. The observed bias may have occurred because, in the learning phase, participants chose *A*_2_ more frequently than *A*_1_. To test this possibility, we ran a logistic regression analysis to see whether the preference of *A*_2_ over *A*_1_ was due to the difference in frequency of choosing *A*_2_ versus *A*_1_ in the learning phase. This analysis showed that the effect on the transfer bias of participants having chosen *A*_2_ more frequently than *A*_1_ in the learning phase was almost significant for the Partial version, but not significant for the Complete version (*t*-test on the regression weights, Partial: *p* = 0.054; Complete: *p* = 0.12). The significant intercept of the regression confirms the transfer effect, even when choice frequency is controlled (*t*-test on the Intercept weight, Partial: *p* = 0.03; Complete: *p* = 0.02). Although the above analysis has been done on the first iteration of (*A*_1_, *A*_2_), the result is almost the same when we consider all iterations of (*A*_1_, *A*_2_), i.e., the rates of choosing *A*_2_ over *A*_1_ (*t*-test on the regression weights, Partial: *p* = 0.0851; Complete: *p* = 0.060, *t*-test on the Intercept weight, Partial: *p* = 0.081; Complete: *p* = 0.080).

Furthermore, we repeated the analysis described in the previous paragraph for the last 20 trials. We again found no significant effect of late choice frequencies on the transfer bias (t-test on the regression weights, Partial: *p* = 0.56; Complete: *p* = 0.29) while intercepts remained almost significant (Partial: *p* = 0.06; Complete: *p* = 0.03). Although the above analysis has been done on the first iteration of (*A*_1_, *A*_2_), the result is the same when we consider all iterations of (*A*_1_, *A*_2_) (*t*-test on the regression weights, Partial: *p* = 0.730; Complete: *p* = 0.798, *t*-test on the Intercept weight, Partial: *p* = 0.132; Complete: *p* = 0.108).

The other possible confounding factors for the transfer bias might be the amount of very small or very large rewards (upper or lower tails of the reward distributions). To test this, first, we summed up the rewards greater than μ + 2.5σ (μ and σ are the mean and standard deviation of the rewards, respectively), and using logistic regression analysis, we tested whether this sum had a significant effect on the transfer bias. We repeated the same analysis for rewards less than μ − 2.5σ. We found no significant effect of large or small rewards in either version (*t*-test on the regression weights, large rewards: [Partial: *p* = 0.40; Complete: *p* = 0.62], Small rewards: [Partial: *p* = 0.54; Complete: *p* = 0.47]). Again, although the above analysis has been done on the first iteration of (*A*_1_, *A*_2_), the result is the same when we consider all iterations of (*A*_1_, *A*_2_) (*t*-test on the regression weights, large rewards: [Partial: *p* = 0.684; Complete: *p* = 0.508], Small rewards: [Partial: *p* = 0.630; Complete: *p* = 0.879]).

Next, we assessed whether the confidence participants reported about their choices differed in the two feedback versions. To this end, we ran a *t*-test analysis and found no significant difference in reported confidences between two feedback versions [*p* = 0.156, *t*_(75)_ = −1.43, *t*-test].

### 2.4. Value Estimation

We then turned our attention to the analysis of the value estimation phase. We found that participant were able to estimate the expected rewards of the advantageous options fairly accurately, but they significantly underestimated the expected rewards of the non-advantageous options (Figure 3C). These results can be explained as follows. When a given option is chosen frequently, participants could either track its average rewards or calculate its value at the moment of estimation.

Our next question was whether the value estimation phase introduced any bias similar to that introduced by the transfer phase. To test this, we ran a paired *t*-test analysis on the estimated values. We found that there was no significant difference between estimation of *A*_1_ and *A*_2_ in either version, yet there was a trend toward overestimating *A*_2_ compared to *A*_1_ [Partial: *p* = 0.1457, *t*_(34)_ = −1.48; Complete: *p* = 0.651, *t*_(41)_ = −0.45; paired *t*-test]. To assess whether there are any differences in estimation variabilities in the two feedback versions, we considered the standard error of the four reported values for each stimulus. To analyze this, we ran a t-test analysis and found that there were no significant differences in estimation variabilities in the two versions [*p* = 0.888, *t*_(75)_ = 0.141, *t*-test].

### 2.5. Comparison Effect

In the next step, we studied the effects of regret and relief on participants' behavior. The idea of regret and relief is that, to learn the consequences of one's decision, one compares the outcome of the selected option with that of the non-selected option. This comparison triggers regret or relief depending on whether the outcome of one's decision is worse or better, respectively, than the outcome of the opposite decision. People naturally tend to avoid regret (approach relief), and when facing regret (relief), they are likely to switch to the other option (or select the same option again; Camille et al., 2004; Coricelli et al., 2005).

In each trial of our experiment, regret and relief were operationalized as the difference between outcomes in that trial. To test whether the difference in outcomes of the previous trial influenced the decision to select a different option (“switch”) or the same option (“stay”) as in the previous trial in the current trial, we used a hierarchical logistic regression analysis as follows. *action* ~ 1 + *vdif* + *odif* + *cond* + (1 + *vdif* + *odif* + *cond*|*subject*), where *action* is the participants switching behavior (1 if participant switched, 0 if participant stayed), and *odif* is the outcome difference of the previous trial and the value difference of the current trial. The outcome difference in the Complete version was defined as the difference between the factual and counterfactual outcomes, {*r*_*FC*_ − *r*_*CF*_}, and for the Partial version, we used *V*_*CF*_ instead of *r*_*CF*_. The *vdif* variable is the option values difference, *cond* variable is a categorical variable with 1 for the *A*_1_*B* pair and 2 for the *A*_2_*C* pair, and *subject* is the random effect variable.

We found a significant comparison effect in the Complete version, but not in the Partial version (Table 1). This means that participants tended to switch from or stay with their previous choice according to whether they were facing regret or relief, respectively, and this tendency was stronger in the Complete version. To investigate this effect more thoroughly, we performed a similar analysis on the logarithm of reaction times: *logrt* ~ 1 + *vdif* + *odif* + *cond* + (1 + *vdif* + *odif* + *cond*|*subject*). We observed that, in the Complete version but not the Partial version, reaction times in each trial were significantly modulated by the outcome difference from the previous trial such that the smaller the difference, the slower the reaction time, and vice versa (Table 1). This result is consistent with the post-error slowing phenomena reported frequently in the decision-making literature (Jentzsch and Dudschig, 2009; Notebaert et al., 2009).

**Table 1:** Comparison effect of the participants' switching behavior and reaction times.

**Switch**									
	**Partial**				**Complete**				
**Name**	**Estimate**	**SE**	**t-stat**	***p*** **-value**		**Estimate**	**SE**	**t-stat**	***p*** **-value**
(Intercept)	−1.5528097	0.10551335	−14.716713	2.69E-48		−2.724902	0.17598005	−15.484153	2.28E-53
Outcome difference	−0.0879529	0.0567055	−1.5510467	1.21E-01		−0.5462195	0.06292942	−8.6798744	4.68E-18
Value difference	−1.123403	0.08767908	−12.812668	3.67E-37		−0.9158512	0.06505058	−14.079062	1.57E-44
Condition	0.25688705	0.08761796	2.93189954	0.00337999		0.25104619	0.12809207	1.95988856	0.05004073
**Reaction time**									
	**Partial**				**Complete**				
**Name**	**Estimate**	**SE**	**t-stat**	***p*** **-value**		**Estimate**	**SE**	**tstat**	**pValue**
(Intercept)	−0.1164283	0.03073684	−3.7879077	0.00015321		−0.1211333	0.03585658	−3.3782727	0.00073263
Outcome difference	0.01123051	0.00651389	1.72408744	0.08473669		−0.0164905	0.00526292	−3.1333433	0.00173402
Value difference	−0.0699353	0.0101347	−6.9005836	5.64E-12		−0.0698999	0.01654412	−4.2250579	2.41E-05
Condition	0.04191482	0.02390541	1.75336139	0.07958424		0.03658956	0.02364193	1.54765513	0.12174177

*The hierarchical logistic regression and hierarchical simple regression analyses were performed on the switching behavior and logarithms of participants' reaction times, respectively. Along with the outcome difference as the main regressor, the current value differences between the two paired options and the condition type (A_1_B, A_2_C) were also included as control regressors. The results show that the participants' current choices as well as their current reaction times were significantly influenced by the outcome differences of their previous choices in the Complete, but not the Partial feedback version*.

### 2.6. Opposing Learning Model (OL)

In the following, we introduce a novel reinforcement learning model, called the Opposing Learning (OL) model, adopted from the standard Q-learning model and inspired by the striatal mechanism. First, we will introduce the basic model for the Partial feedback version, and then we will extend the model for the Complete feedback version.

#### 2.6.1. Model Description

**Figure 4:**
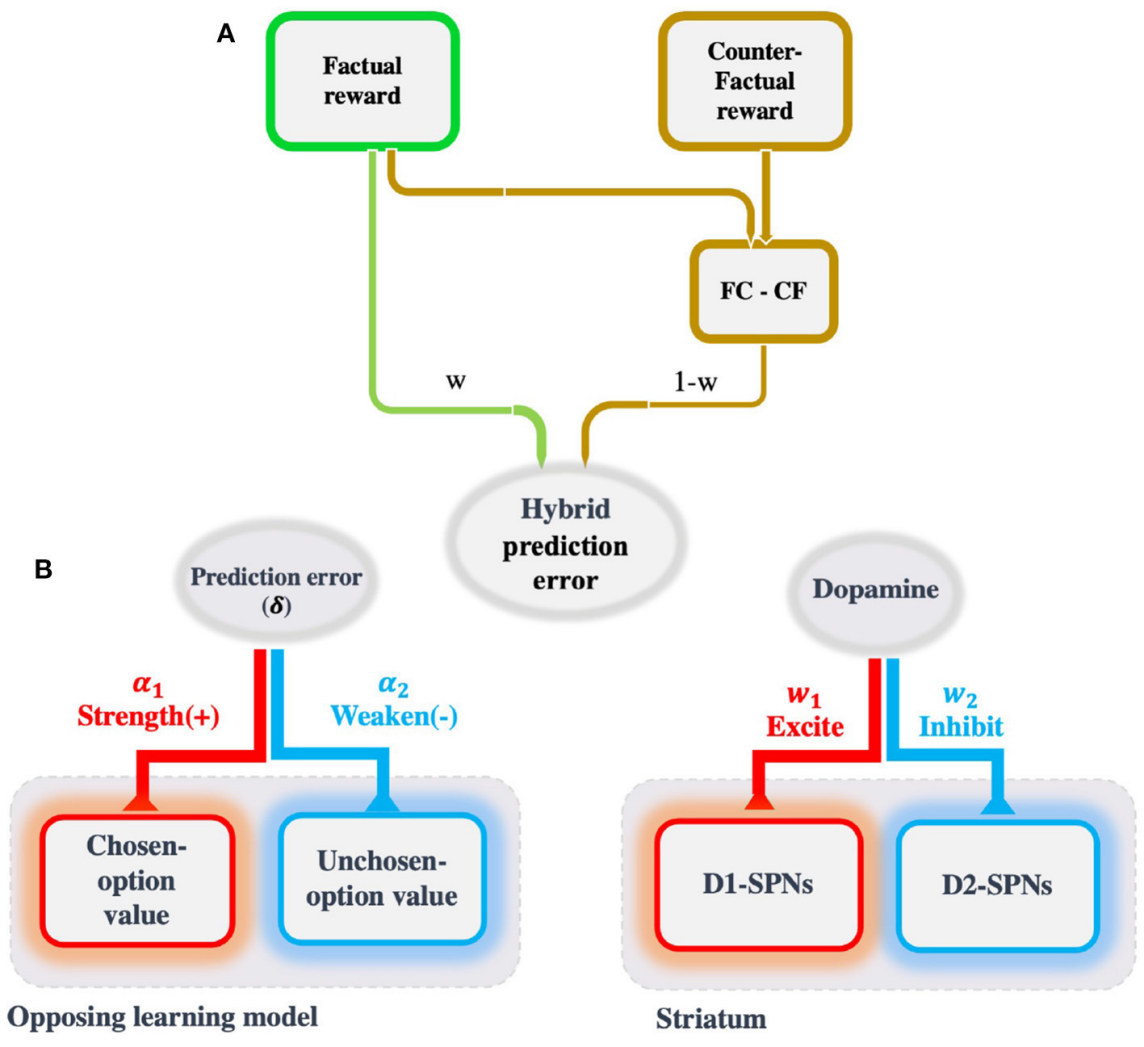
The schematic of the OL model and its extension. **(A)** A common strategy in the value learning task, especially when counterfactual outcomes are also provided, is to compare competing outcomes. This comparison triggers the regret (relief) that subsequently drives avoidance (approach) behavior. The tendency to minimize regret (and maximize relief) along with the tendency to maximize expected rewards, which is a hybrid strategy, can better account for the behavioral data than either of these strategies. The absolute and relative weights assigned to each strategy (maximize expected rewards and minimize regret) determine the amount of their effect on behavior. **(B)** The idea behind the OL model comes from the opposing effect of dopamine on two distinct populations of spiny projection neurons (viz., D1 and D2). It has been proposed that they encode the values of chosen and unchosen options, respectively, by promoting the latter and inhibiting the former. Similarly, in the inspired model, chosen prediction error has an opposing role in updating the chosen and unchosen option values, by strengthening the latter and weakening the former.

Our model focuses on the chosen option in the sense that value updating is based solely on the prediction error of the chosen option. Following the choice, the chosen prediction error will simultaneously update the chosen and unchosen values in opposite directions (increasing and decreasing, respectively).


$$Q_{ch}=Q_{ch}+\alpha_{1}\delta_{ch}$$

$$Q_{un}=Q_{un}-\alpha_{2}\delta_{ch}$$

where *ch* refers to the *chosen* option, *un* refers to the *unchosen* option, and δ_*ch*_ = *r*_*ch*_ − *Q*_*ch*_. At the final stage, the decision is made following the softmax rule, $p\left(c\right)=\frac{1}{1+e^{\beta \left(Q_{un}-Q_{ch}\right)}}$, where β is the inverse of the temperature parameter. The model equation is inspired by the effect of dopamine on the striatum. The striatum consists of D1 and D2 spiny projection neurons (SPNs) which encode chosen and unchosen values, respectively. The presence of prediction error in both chosen and unchosen value updating comes from the fact that the dopamine release is diffusive and thus non-selective. The specified signs of prediction error in the model equations relates to the opposite effects of dopamine on D1- and D2-SPNs (Figure 4).

#### 2.6.2. Contextual Effect in the OL Model

**Figure 5:**
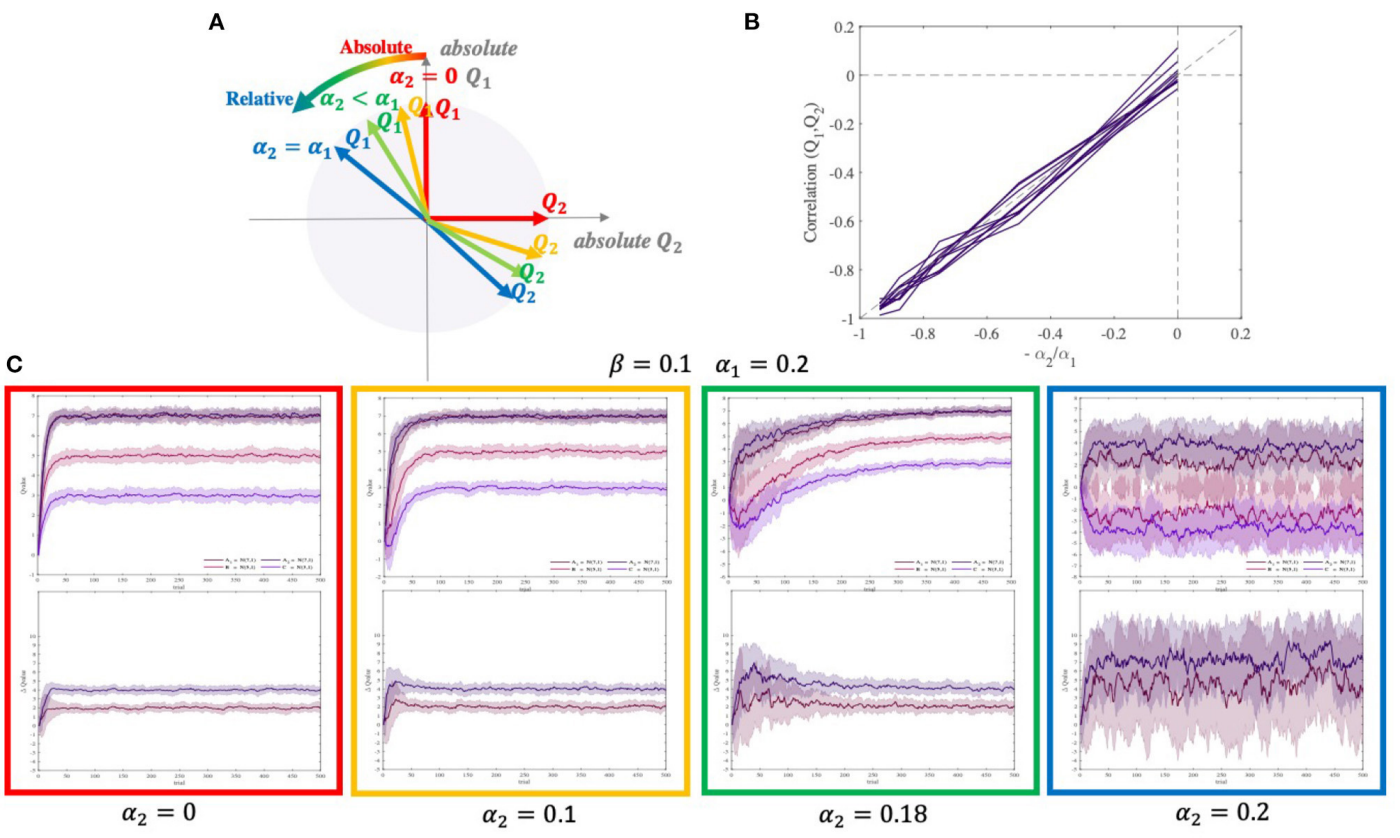
Correlation between two competing option values estimated by the OL model. **(A)** When α_2_ = 0, two estimated values are equal to their absolute values and they are orthogonal. However, whenever α_2_ gets closer to α_1_, the estimated values for each pair become more correlated. Moreover, when α_2_ = α_1_, estimated values are approximately fully correlated(*corr* ≈ −1). **(B)** Correlation between two paired option values as a function of −α_2_/α_1_. **(C)** The difference in the estimated values of A1 and A2 (contextual bias) emerges with increasing α_2_. The diagram for *q*-values and differences of *q*-values have been shown at the top and bottom, respectively. The simulation was performed using two different pairs of options [N(7, 1), N(5, 1)], and [N(7, 1), N(3, 1)], with β = 0.1, α_1_ = 0.2, and four different α_2_ = 0, 0.1, 0.18, 0.2.

In the OL model, the chosen and unchosen values are coupled and thus not independent. We measured the correlation between these two values in a simulation. The correlation turned out to be negative and proportionate to the ratio of two learning rates (Figure 5B):


$$Corr\left(Q_{1},Q_{2}\right)\approx -\frac{\alpha_{2}}{\alpha_{1}}$$

When α_2_ changes from 0 to α_1_, the correlation between *Q*_1_ and *Q*_2_ changes from 0 to −1, and the encoding changes from almost fully absolute to almost fully relative. Figure 5A shows how *Q*_1_ and *Q*_2_ start to move away from orthogonality to fully negatively correlated. In simulations, typical agent α_2_ = 0 shows no contextual effect, agent 0 < α_2_ < α_1_ shows a moderate and temporary contextual effect, and agent α_2_ = α_1_ shows a large and permanent contextual effect (Figure 5C).

#### 2.6.3. Performance of the OL Model

**Figure 6:**
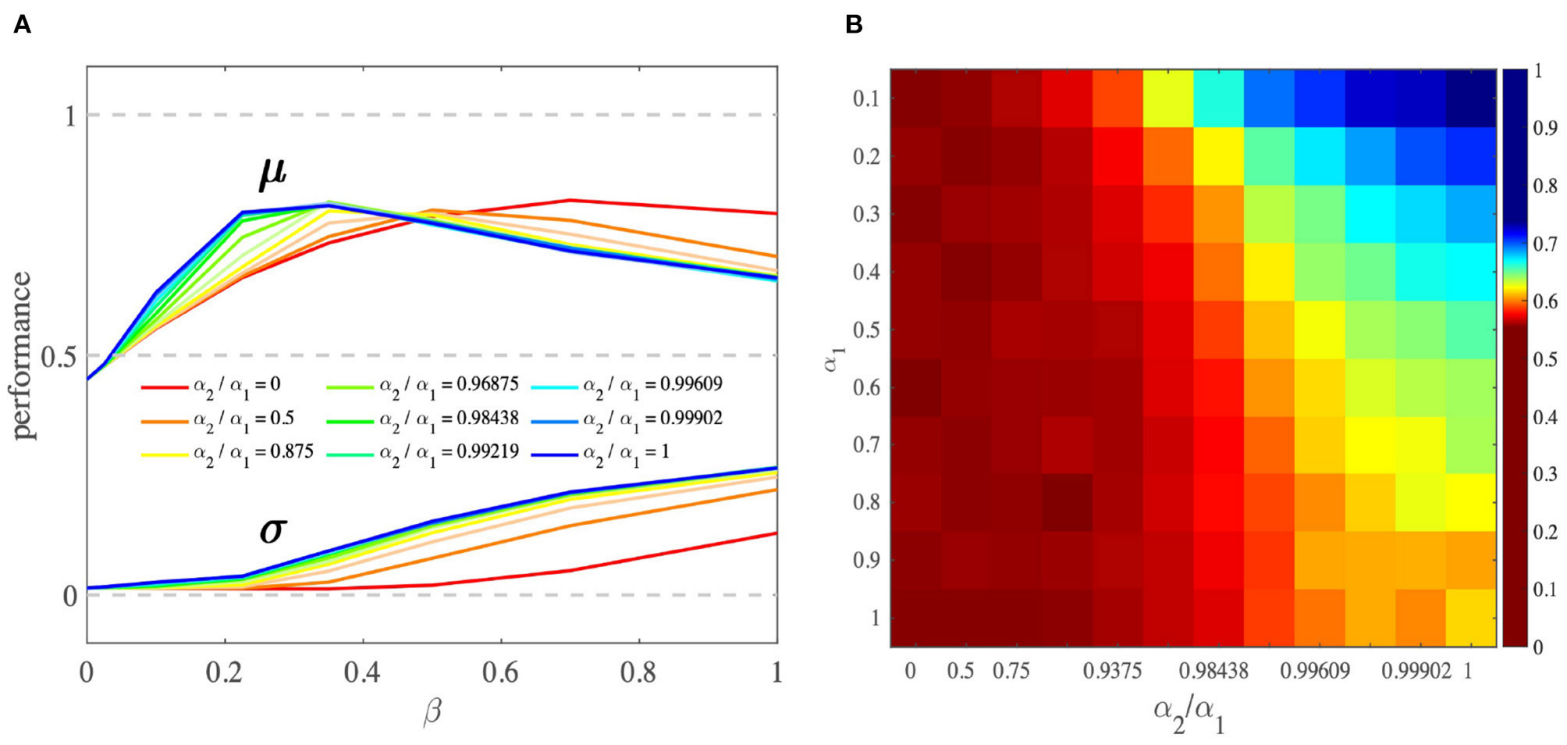
Comparison of OL and SQL model performance. **(A)** As α_2_/α_1_ goes from 0 (SQL) to 1 (the OL_1_), the peak of the performance shifts to the left, where the value of β is smaller and more reasonable. In this β range, performance peaks where α_2_/α_1_ is higher. The larger β is, the larger the behavioral variances. The performances were obtained by averaging performances across all task settings and different ranges of α_2_/α_1_. **(B)** This heat map shows that, by increasing α_2_/α_1_, performance increases. This simulation was performed using two different pairs of options [N(10, 1), N(7, 1)] with β = 0.1. μ and σ stand for the mean and standard deviation of the performance, respectively.

We performed a simulation analysis to study the behavior of the OL model. First, we found that the OL model as a reinforcement learning model performs better when the difference between competing option values increases (Supplementary Figure 2). Second, we studied the effect of parameter α_2_ on agents' learning performance. This analysis showed that when α_2_ > 0, average performance is better than when α_2_ = 0 (SQL model). Moreover, increasing α_2_ results in an increase in average performance (Figures 6A,B). This increase is due to the inhibition role of the chosen prediction error on the unchosen value that would lead to an increase in the contrast between two competing option values, and thus an increase in performance (Figure 6A). Note that the above results are restricted to the case in which the parameter β is in a reasonable range. (For details about the simulation, see Section 4.4).

#### 2.6.4. Extending the OL Model

Several studies have shown that, in the Complete feedback version of the task, in the presence of counterfactual outcomes, the quantity encoded by dopamine is not the simple prediction error alone, but rather a combination of the simple prediction error and the counterfactual prediction error (i.e., the prediction error of the outcome difference; Kishida et al., 2016). Furthermore, some studies have shown that by incorporating the outcome difference into the learning procedure, the model can better account for the behavioral (Bavard et al., 2018) and physiological (Coricelli et al., 2007) data. To this end, we replaced the reward term with a hybrid combination of the absolute reward (*r*_*FC*_) and the relative reward (*r*_*FC*_ − *r*_*CF*_, the outcome difference; Figure 4B). Recall that the outcome difference played a significant role in the participants' switching behavior in the Complete feedback version (see Section 2.5). The updating equations of the extended OL model are exactly the same as those in the original OL model, but the prediction error is defined as follows:


$$\delta_{ch}=r_{hyb}-Q_{ch}$$

$$r_{hyb}=wr_{abs}+\left(1-w\right)r_{rlt}$$

$$r_{abs}=r_{FC},\;r_{rlt}=r_{FC}-r_{CF}$$

where *w* is the weight of the absolute strategy.

It turns out that this extended model becomes an instance of the original model by changing the mean rewards (μ_1_ and μ_2_) to $\mu_{1}^{\prime }=\mu_{1}+\left(1-w\right)\mu_{2}$ and $\mu_{2}^{\prime }=\mu_{2}+\left(1-w\right)\mu_{1}$. Note that since $\mu_{1}^{\prime }-\mu_{2}^{\prime }=w\left(\mu_{1}-\mu_{2}\right)$, the extended OL model is like a simple OL model in which the means have gotten closer to each other. Thus, this modification does not change the main characteristics of the OL behavior, and the extended OL model still preserves all of the above-mentioned properties. This shows how, by designing a proper prediction error, the OL model can be successfully extended for a wide range of conditions.

### 2.7. Model Comparison

#### 2.7.1. Model Fitting and Model Validation

In this part of the analysis, we ran model comparison analyses in two ways: model fitting (learning phase) and model validation (transfer phase). The models we used in our model space consists of some models as benchmarks and some models that aim to explain context-dependent value learning. Our main model-space included the standard Q-learning model (SQL), the reference-point model (RP) (Palminteri et al., 2015), the difference model (Klein et al., 2017), and the hybrid model (Bavard et al., 2018). The same analysis was also performed on the extended model-space which, in addition to the previously named models, included the forgetting reinforcement learning model (FQL) (Barraclough et al., 2004; Ito and Doya, 2009; Katahira, 2015; Niv et al., 2015; Kato and Morita, 2016), the experienced-weighted attraction model (EWA) (Camerer and Hua Ho, 1999), the sample-based episodic memory model (SBE) (Bornstein et al., 2017), and RelAsym model (Garcia et al., 2021; Ting et al., 2021, Supplementary Tables 2–4).

Except for the difference model, which only had the Complete version, all of the models had two Partial and Complete feedback versions. The OL model had two different versions, OL_1_ in which the chosen and unchosen options had the same learning rates, and OL_2_ in which they had different learning rates. For the details of the models, see Section 4.

For the learning phase, we performed the fitting procedure for each participant and each model separately, and calculated their exceedance probabilities (xp). For the transfer phase, we calculated the negative log-likelihood for the all iterations. Through model comparison, we found that the OL_1_ model (for the Partial and Complete versions), fit the data better in the learning phase and also predicted the data better in the transfer phase (Table 2). In addition to the model fitting analysis, we applied all of the behavioral analysis in the Performance and Contextual effect sections on the simulated data. The simulation for each participant in each model was conducted by the participant's best-fitted parameters (averaged over 100 repetitions).

**Table 2:** Model comparison: model fitting and model prediction.

	**SQL**	**RPA**	**Dif**	**Hyb**	**OL_1_**	**OL_2_**
**FITTING (LEARNING PHASE)**						
**Partial**						
xp	2***e***−05	0		0	0.99998	0
pxp	2.0047***e***−05	4.7129***e***−08		4.7129***e***−08	0.99998	4.7129***e***−08
**Complete**						
xp	0.001594	0	0.16604	0.000685	0.66409	1***e***−06
pxp	0.0024225	0.00083783	0.16591	0.0015188	0.66104	0.00083883
**PREDICTION (TRANSFER PHASE)—ALL ITERATIONS**						
**Partial**						
***A***_**1**_ ***A***_**2**_	2.77 ± 0.16	2.83 ± 0.22		2.88 ± 0.21	2.51 ± 0.14	2.63 ± 0.13
all	9.15 ± 0.55	9.05 ± 0.52.		9.27 ± 0.53	8.99 ± 0.63	9.12 ± 0.6
**Complete**						
***A***_**1**_ ***A***_**2**_	4.69 ± 0.84	4.8 ± 0.85	4.2 ± 0.75	4.2 ± 0.64	3.49 ± 0.44	3.5 ± 0.45
all	15.42 ± 2.82	14.11 ± 2.01	12.88 ± 1.91	14.06 ± 1.86	12.26 ± 2.05	12.27 ± 1.85

*Fitting: Bayesian exceedance probability (xp) (Stephan et al., 2009) and protected exceedance probability (pxp) (Rigoux et al., 2014) of the learning phase. Prediction: negative log-likelihood (nll) of A_1_A_2_ and all six combinations of the transfer phase separately. Mean±SEM*.

This analysis showed that the *OL*_1_ model was able to generate all key signatures of the behavioral data (Figures 3A,B). In the learning phase, agents' performances were higher than 0.5 (Partial: performance = 0.6637±0.0627; Complete: performance = 0.8857±0.0639; Figure 3A), and the performance in the learning phase of the Complete version was significantly higher than that in the Partial version [*p* = 4.4086*e* − 25, *t*_(75)_ = 15.3079, one-tailed *t*-test]. We also observed that agents' performance was significantly better than random (0.5) in the transfer phase [Partial: performance = 0.8238±0.1429, *t*-test, *p* = 3.2597*e* − 22, *t*_(34)_ = 22.3594; Complete: performance = 0.9587±0.0746, *t*-test, *p* = 1.2272*e* − 34, *t*_(41)_ = 39.6801; Figure 3B, Supplementary Figure 1]. We were also able to replicate the transfer effect (Figure 3B): Agents preferred *A*_2_ over *A*_1_ in both feedback versions (Partial: *p* = 0.04096, *ratio* = 0.65714; Complete: *p* = 6.8771*e* − 05, *ratio* = 0.78571; binomial test).

We next assessed how the estimated parameter β is different across feedback versions. To do so, we ran a *t*-test analysis and found that the exploitation rate β was significantly higher in the Complete version than in the Partial version (partial: *mean* = 0.0705, complete: *mean* = 0.368, *p* = 1.085*e* − 07, *t*-test). Thus, participants explored less in the Complete version than in the Partial version.

#### 2.7.2. Parameter Recovery and Model Recovery

To validate our model fitting and model comparison procedures, we conducted parameter recovery and model recovery analyses, respectively (Correa et al., 2018; Wilson and Collins, 2019).

To do these analyses, using a common approach in the literature (Daw et al., 2011; Palminteri et al., 2015; Correa et al., 2018), we fitted beta distributions to the best fitted parameters of all participants. Then we sampled synthetic participants from these distributions. Then we generated 30 × *numberofsubjects* simulated behaviors with all models in the main model space (30 repetitions resulting 30 × 35 simulations for the Partial version, and 30 × 42 simulations for the Complete version). Then we fitted the generated data by each model in the main model space to find which models best fitted to these generated data. It should be noted that the task configurations were the same as those used for the real participants.

**Figure 7:**
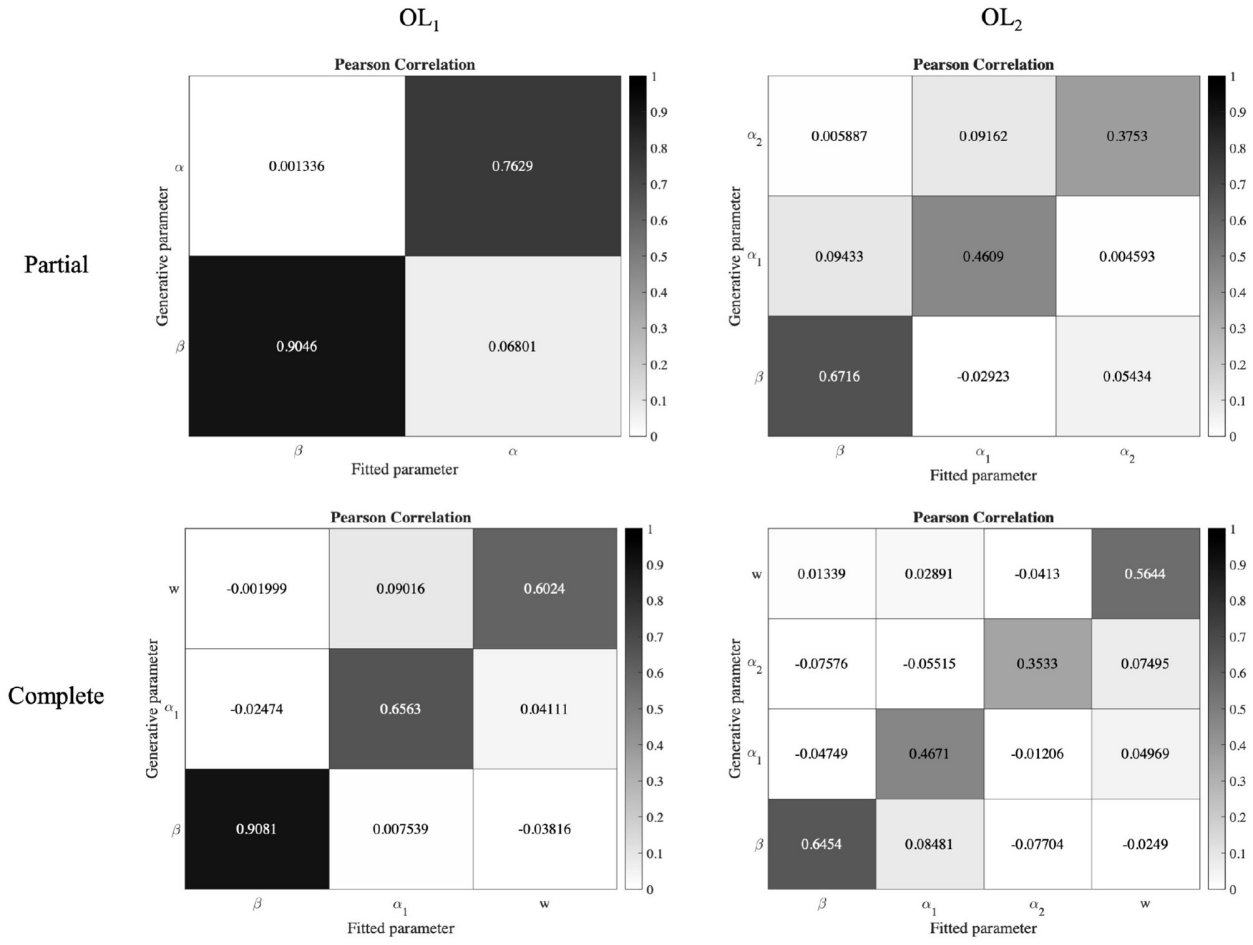
Parameter recovery analysis of the OL models. Data from 30 × *number of subjects* were simulated with the OL models. The Pearson correlation between the true and recovered parameters of the OL models shows they have strong correlations.

For parameter recovery analysis, from the above simulation data we took the generated and fitted parameters of the OL models, and calculated the Pearson correlation of them. As can be seen in the Figure 7, the correlations between fitted and recovered parameters are strong. We also regressed recovered parameters against the true parameters. The result of the regression has been reported in the Table 3, and shows an acceptable parameter recovery.

**Table 3:** Parameter recovery of the *OL*_1_ model: regression results.

	**Coef**	**Parameter**	**Estimate**	**SE**	***p*** **-value**		**Parameter**	**Estimate**	**SE**	***p*** **-value**
										
	*b*_0_	β	0.004	0	0.00E + 00		β	0.005	0.001	0
	*b*_1_		0.818	0.013	0			0.865	0.016	0
										
Partial	*b*_0_	α	0.038	0.004	0.00E + 00	Complete	α	0.058	0.004	0
	*b*_1_		0.734	0.026	0.00E + 00			0.611	0.025	0
										
	*b*_0_						*w*	0.143	0.007	0
	*b*_1_							0.549	0.021	0
										

*The recovered parameters were regressed against the true parameters. The results of the intercepts (b_0_) and slopes (b_1_) showed an acceptable parameter identification*.

In the model recovery analysis, our aim is to investigate whether the models in the model space can be distinguished from each other. To do this, we used the model recovery approach in the paper of Wilson and Collins (Wilson and Collins, 2019; Ciranka et al., 2022). According to this approach we calculated two metrics: the conditional probability that a model fits best given the true generative model [*p*(*fit*|*gen*)], and the conditional probability that the data was generated by a specific model, given it is the best fitted model [*p*(*gen*|*fit*)]. To calculate *p*(*fit*|*gen*), we took the fitted data on our generated datasets and calculated the corresponding AICs to see how often each model provided the best fit. To calculate *p*(*gen*|*fit*), we used the following Bayes formula with the uniform prior over models *p*(*gen*):


$$p(gen⏐fit)=\frac{p(fit|gen)p(gen)}{\sum_{m=1}^{nmodels}p{\left(fit|gen\right)}_{m}p{\left(gen\right)}_{m}}$$

**Figure 8:**
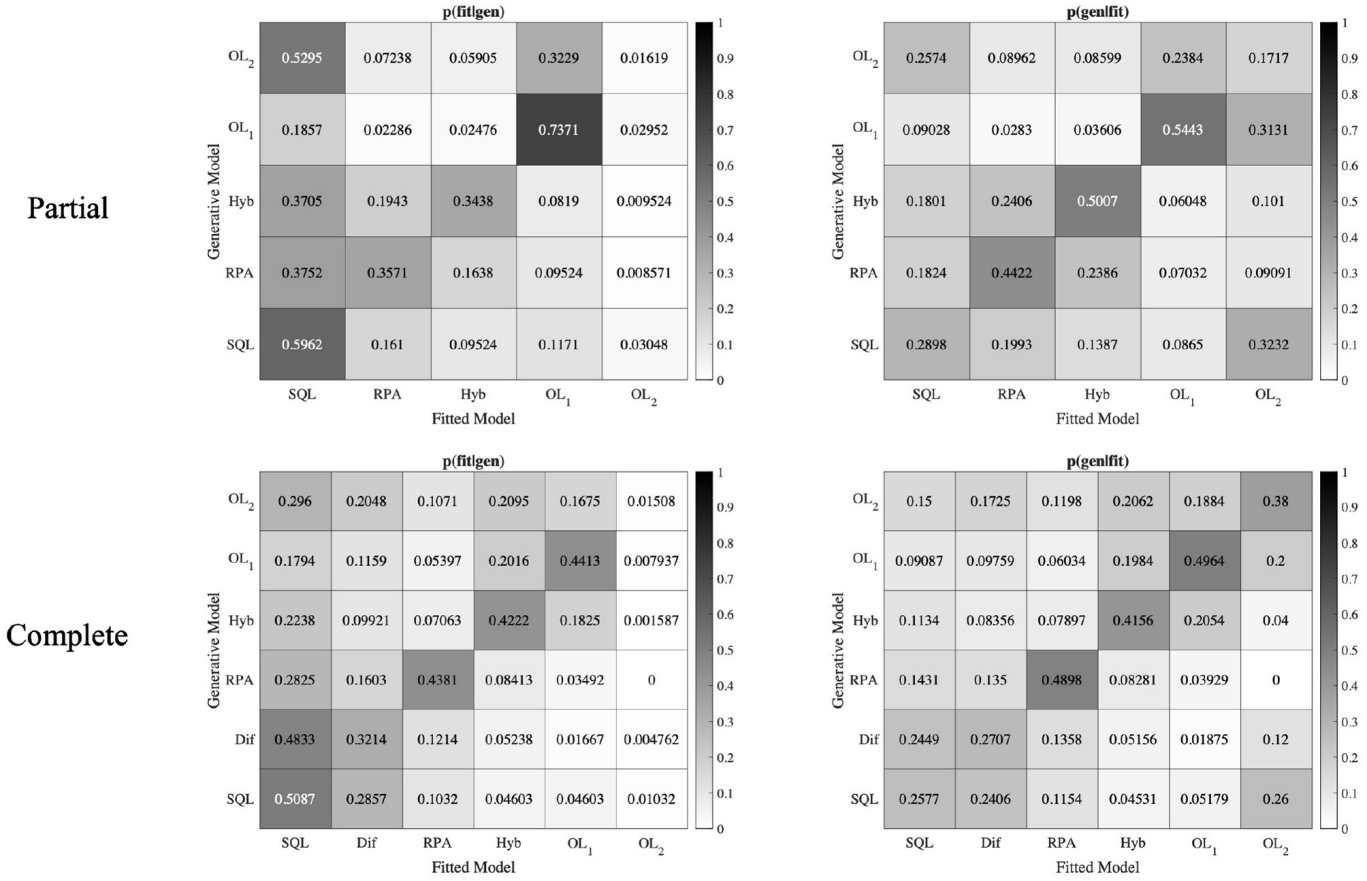
Model recovery analysis. Data from 30 × *number of subjects* were simulated with all models in the model space. The generated data were fitted by all models in the main model-space. The *OL*_1_ model (in the Partial and Complete versions) could be strongly identified.

If we could recover our models perfectly, the *p*(*fit*|*gen*) matrix must be an identity matrix (a matrix with all the diagonal entries 1 and other entries 0). Unfortunately, some of the models in our model space have rather similar behavior on this task (e.g., the Hybrid model with *w* = 1 is identical to the *SQL* model), therefore we have large off-diagonal elements in this matrix (Figure 8). Since the model recovery was not perfect, we conducted *p*(*gen*|*fit*) analysis, which is a more critical metric to investigate model recovery analysis (Wilson and Collins, 2019; Ciranka et al., 2022). As can be seen in the Figure 8, in the Partial version, all diagonal entries of the *p*(*gen*|*fit*) matrix, except *OL*_2_ are dominant in their columns which shows that all the models except *OL*_2_ could be identified well. In the Complete version, all diagonal entries of the *p*(*gen*|*fit*) matrix, except *SQL* and *Dif* models are dominant in their columns. This analysis shows that all the models could be distinguished from each other, except *SQL* model which could not be confidently distinguished from *Dif* model.

We conducted model recovery analysis to identify *OL*_2_ model with a specific range for α_2_ parameter (α_2_ is close but not equal to α_1_), and it was successfully identified. Unfortunately by using the range of best fitted parameters to the behavioral data, *OL*_2_ model could not be recovered. It is critical to note that, although some models could not be identified well, our newly introduced model *OL*_1_ that is also the winning model in the model-comparison procedure, could be significantly recovered and we can see no strong mixing behavior between *OL*_1_ and other models.

## 3. Discussion

Studies of the contextual effect on value learning have mostly focused on the putative role of the unchosen outcome in updating the chosen value in the Complete feedback paradigm (Palminteri et al., 2015; Klein et al., 2017; Bavard et al., 2018). In this study, we showed that we are able to explain the contextual effect in the Partial feedback paradigm by using the chosen outcome in updating the unchosen value. Inspired by the opposing effect of dopamine in the striatum on competing option values, we introduced the novel Opposing Learning model, in which the chosen prediction error updates the chosen and unchosen values in opposing directions. This update rule will make the competing option values correlated to each other, which leads to the emergence of the contextual effect during value learning. On the other hand, due to the inhibitory role of the prediction error in updating unchosen values, the contrast between option values compared to the standard Q-learning model will increase, which leads to a higher performance average. Compared to previous models, this model was better able to account for the behavioral characteristics of the data (Camerer and Hua Ho, 1999; Palminteri et al., 2015; Kato and Morita, 2016; Bornstein et al., 2017; Klein et al., 2017; Bavard et al., 2018; Sutton and Barto, 2018).

Most studies on the instrumental learning paradigm use discrete rewards (1 and 0) as gain and loss. Participants then estimate the probability of rewards for each option to maximize their payoffs (Frank et al., 2004; Palminteri et al., 2015; Klein et al., 2017). Although we sometimes encounter probabilistic rewards in our daily lives (e.g., probability of making a profit on a stock, at a horse race), we more often experience continuous outcomes of our choices, as in the amount of profit from a financial transaction (e.g., stocks, pension plans) or evaluation metrics (assessment scores, citation indices or any other case with quantitative outcomes) and estimate the magnitude of our expected outcomes based on these continuous outcomes. Therefore, our secondary aim in this study was to investigate the contextual effect in a paradigm with continuous reward magnitude. We adapted previous instrumental learning tasks with novel reward designs, in which the stimuli were associated with some rewards drawn from specific normal distributions. With these complementary results, we showed that the contextual effect is not limited to probabilistic rewards, but extends to magnitude rewards.

There are two pathways in the basal ganglia with opposing roles: the direct pathway, which promotes actions, and the indirect pathway, which suppresses actions (Cox and Witten, 2019; Peak et al., 2019). These pathways originate from two distinct populations of striatal neurons, D1- and D2-SPNs, on which dopamine has an opposing effect (viz., stimulating D1-SPNs and inhibiting D2-SPNs; Surmeier et al., 2007; Shen et al., 2008). Associative learning studies have shown that D1- and D2-SPNs encode the values of the chosen and unchosen options, respectively (Frank et al., 2004; Tai et al., 2012; Collins and Frank, 2014; Donahue et al., 2018; Nonomura et al., 2018; Shin et al., 2018; Bariselli et al., 2019). Inspired by these results, we introduced a novel model in which the chosen prediction error updates the chosen and unchosen values concurrently, but in an opposing manner (the latter with positive and the former with negative coefficients). The only model in the literature with similar update rules is the OpAL model introduced by Collins and Frank (2014). The crucial difference between the OpAL and OL models is that, while the OpAL model uses a reference-point mechanism to account for the contextual effect, the OL model can better explain the effect without resorting to the concept of reference point.

The parameter in the OL model that controls the magnitude of the correlation between competing option values (as an indicator of the contextual effect) is α_2_. According to whether α_2_≈0, α_2_≈α_1_, or 0 < α_2_ < α_1_, there are three regimes. When α_2_≈0, the correlation is at its lowest (*corr*≈0) and there is no contextual effect at all. When α_2_ = α_1_, the absolute correlation is at its highest (*corr*≈ − 1) and the contextual effect is the strongest and permanent. Finally, when 0 < α_2_ < α_1_, the correlation is moderate and the contextual effect is moderate and temporary, disappearing over time (Figure 5). This negative correlation between the chosen and unchosen values in the OL model (especially in the *OL*_1_ model) causes the competing option values to be learned relative to each other (*q*_*un*_≈ − *q*_*ch*_). By this relative encoding, this model can explain not only the reward learning behavior but also the punishment avoidance learning behavior (Palminteri et al., 2015; Palminteri and Lebreton, 2021).

The average performance of the OL model is better than that of the SQL model. In environments with a reasonable amount of noise, the more relative the model (α_2_ closer to α_1_), the better the average performance. The performance of the OL model improves as a result of increased contrast between option values, which makes detection of the superior options easier. We should also mention some other related models. First, the confirmation bias model (Lefebvre et al., 2022) which improves the performance in the same way. In this model, it is the asymmetric updating of positive and negative prediction errors that improves the performance by increasing the contrast between option values. Second, the RelAsym model (Garcia et al., 2021; Ting et al., 2021) which is the combination of the confirmation bias and reference point mechanisms. The RelAsym model by having these two factors, not only has the asymmetric updating advantage (performance advantage) but also is able to explain the contextual effect because of the reference point function it used in its mechanism. The RelAsym model from the performance's and contextual effect's viewpoints is similar to the OL model, but these two models are different in their main underlying mechanisms. The RelAsym model uses the explicit reference point mechanism to explain the contextual effect, while the OL model can explain the contextual effect without using any explicit reference point mechanism.

One of the advantages of the OL model is that it can be extended for the Complete feedback version. Several studies have shown that people performing the Complete version of the task are affected not only by absolute rewards (chosen outcomes), but also by relative rewards (the difference between chosen and unchosen outcomes; Camille et al., 2004; Coricelli et al., 2005, 2007). These relative rewards are encoded in the brain by dopamine (Kishida et al., 2016; Lak et al., 2016). Our results are consistent with these findings. In Section 2.5, we showed that relative rewards have a significant effect in the Complete version, but not in the Partial version (Table 1). This suggests that participants are using a hybrid strategy, that is, a weighted combination of absolute and relative rewards, when performing the Complete version. This finding is similar to those of previous studies (Coricelli et al., 2007; Bavard et al., 2018). It is noteworthy that the extended OL model preserves all the essential characteristics of the basic OL model.

There are two types of learning models in which the unchosen values are updated when people observe the chosen rewards. The “reference-point learning model” is an example of the first type. In this model, the reference point of a state, which is equivalent to its expected rewards, is updated continuously with its outcomes. The valences of its outcomes are specified relative to the reference point. The valence is positive when the outcome is greater than the reference point and negative when the outcome is smaller than the reference point. Thus, in the first type, the values of the competing options are learned relative to each other (Palminteri et al., 2015; Klein et al., 2017; Bavard et al., 2018).

In the second type, the competing values are learned independent of each other. The “forgetting reinforcement learning model” is an example of the second type. Despite similarities between the OL model and the forgetting reinforcement learning model, there are crucial differences between them. First, in a forgetting reinforcement learning model, the unchosen value decays over time. Therefore, if an option has not been chosen for a long time, its value decays toward zero. However, in the OL model, the unchosen value does not decay, but is updated by the chosen prediction error in an opposing direction. This implies that, if an option has not been chosen for a long time, its value does not decay to zero, but converges toward [ − α_2_/α_1_×chosen value]. Second, in contrast to the OL model, in the forgetting reinforcement learning model, the observed rewards of the chosen options do not affect the values of the unchosen options, so the competing values are learned independently of each other.

Taken together, we have shown that context affects people's behavior even in everyday conditions when there is no counterfactual outcome available. Although this contextual effect leads to an ecological advantage by allowing one to gain more rewards in the original context, it results in suboptimal decision making outside the original context. Studying the mechanism underlying context-dependent behavior can also help us to find a solution for the problems that might arise from suboptimal behavior.

## 4. Materials and Methods

### 4.1. Participants

Two groups of 41 and 47 participants took part in the Partial and Complete versions of the experiment, respectively. We excluded six participants from the Partial version and five participants from the Complete version. In the Partial and Complete versions, two and three participants, respectively, did not learn the associations, and the difference of expected rewards for *A*_1_ and *A*_2_ exceeded one for four and two participants, respectively. After their exclusion, *N* = 35 participants [age: 26±6 (*mean*±*SD*), female: *n* = 16] and *N* = 42 participants [age: 23±5 (*mean*±*SD*), female: *n* = 12] remained for the Partial and Complete versions, respectively. They received their monetary rewards according to their performance after completing the task. They were all healthy volunteers that gave written informed consent before starting the task. The study was approved by the local ethics committee.

### 4.2. Behavioral Task

Two different cohorts of participants performed two different versions of instrumental learning tasks, which had been adapted from previous studies (Palminteri et al., 2015; Klein et al., 2017; Bavard et al., 2018). The two tasks were structured very similarly and included three consecutive phases of learning, post-learning transfer, and value estimation. The tasks differed with respect to the way feedback was provided to the participants. In the Partial version of the task, only the outcomes for the chosen option (factual outcomes) were provided to the participants; in the Complete version, both the outcomes for the chosen and unchosen options (factual and counterfactual outcomes) were provided. Before the main task, participants performed a short training session (20 trials) to become familiarized with the learning phase. The stimuli and the reward statistics of the training session were different from those of the main session. The stimuli were selected from the Japanese Hiragana alphabet.

The learning phase was made up of one session in which, in each trial, two stimuli were presented on the screen, and participants were instructed to choose the option with the higher expected reward. This instrumental learning paradigm resulted in participants gradually learning, through trial and error, to choose the most advantageous option in each trial. The cues were shown to the participants from two pairs of stimuli {*A*_1_*B, A*_2_*C*}, which means that, in each pair, each stimulus was always presented with a specific other stimulus. Each stimulus pair thus established a fixed context. These two contexts were pseudorandomly interleaved across trials. The rewards of *A*_1_ and *A*_2_ stimuli were drawn from the same normal distribution of N(64, 13) and the rewards of *B* and *C* stimuli were drawn from different normal distributions of N(54, 13) and N(44, 13), respectively. To control for confounding factors, reward samples were drawn from the truncated distribution, which was in the [μ − 3σ, μ + 3σ] ([0, 100]) interval. The parameters of the distributions were unknown to the participants, and they were supposed to learn them. Although the reward statistics of *A*_1_ and *A*_2_ were the same, the images associated with them were different to conceal the task structure from the participants.

The side on which each stimulus was presented on the screen, whether to the right or left of the fixation point, was also pseudorandomized during the task, such that for the total number of trials for each context, a given stimulus was presented on the right in half of the trials and on the left in the other half. The participants were asked to select their choices within 4,000 ms. Otherwise they missed the reward in that trial and the “No Response” message was shown on the screen. In each trial, the participants selected their choice by pressing the left or right arrow key for the options displayed on the left or right, respectively. Following the choice, the chosen option was surrounded by a blue square and the related outcomes were presented simultaneously on the screen. In the Partial version, the factual outcome was shown below the chosen option for 500 ms. In the Complete version, both the factual and counterfactual outcomes were shown below the chosen and unchosen options for 1,000 ms, respectively. In the Complete version, participants were to process twice the amount of information processed in the Partial version. In our pilot study, we found that having only 500 ms to process two continuous outcomes was not sufficient and resulted in poorer performance in the Complete compared to the Partial version, so we increased the presentation time in the Complete version to 1,000 ms. The next trial started after a 1,000-ms fixation screen. Each context was presented to the participants in at least 50 trials for a total of at least 100 trials. After at least 100 trials, the task continued for each participant until the experienced mean of *A*_1_ became almost equal to the experienced mean of *A*_2_ (i.e., their difference became <1). If this condition was not met by the 300th trial, the learning phase was stopped and the participant's data were excluded analysis. By this design, the number of trials always fell into the range of [100, 300] and the number might be different for each participant.

After the learning phase, participants immediately entered the post-learning transfer phase. We did not inform them about the transfer phase until they had completed the learning phase, so that they would not use any memorizing strategies during the learning phase. In the transfer phase, all possible binary combinations of the stimuli (six combinations) were presented to the participants and they were asked to choose the option with higher expected rewards. We informed them that, in the transfer phase, they would not only see previously paired options, but also options that had not been paired in the preceding (learning) phase. Each combination was presented four times, giving a total of 6 × 4 = 24 trials that were presented in a pseudorandomized order. In contrast to the learning phase, the transfer phase was self-paced (i.e., participants were not forced to choose within a limited time) and no feedback was provided to the participants in order not to interfere with their learned values (Frank et al., 2004, 2007; Palminteri et al., 2015; Klein et al., 2017; Bavard et al., 2018). Following each choice, using the computer mouse, participants were to report their level of confidence about their choice on a scale of 0–100, whereby the left side of the axis was labeled “completely unsure” and the right side “completely sure.”

After the transfer phase, participants completed the value estimation phase. In the value estimation phase, stimuli were presented to the participants one by one. Participants were asked to estimate average rewards on a scale of 0–100. Each stimulus was presented four times giving a total of 4 × 4 = 16 trials which were presented pseudorandomly. These trials were also self-paced and no feedback was provided to the participants. We informed the participants that their payoff would be based on the sum of rewards they earned during the learning task. In the Complete version, the participants' total rewards were based solely on the rewards of their choices. Although they were not paid in the transfer and value estimation phases, they were encouraged to respond as correctly as possible as if their rewards depended on correct responses. At the end of the task, their total rewards were shown on the screen.

### 4.3. Computational Models

#### 4.3.1. The Standard Q-Learning (SQL) Model

Context-dependent learning models are commonly compared to the standard Q-learning (SQL) model as a benchmark (absolute learning model). In the SQL model, the value of each option is updated only based on its own outcomes (Sutton and Barto, 2018).


$$\delta_{ch}=r_{ch}-Q_{ch}$$

$$Q_{ch}=Q_{ch}+\alpha \delta_{ch}$$

In the simplest form, only the chosen option is updated based on its outcomes, while in the extended form the unchosen options are also updated, but again with their own outcomes:


$$\delta_{ch}=r_{ch}-Q_{ch}$$

$$Q_{ch}=Q_{ch}+\alpha_{1}\delta_{ch}$$

$$\delta_{un}=r_{un}-Q_{un}$$

$$Q_{un}=Q_{un}+\alpha_{2}\delta_{un}$$

In this model, the learning rates can be the same or different (α_1_ = α_2_ or α_1_≠α_2_).

#### 4.3.2. The Reference-Point (RP) Model

The idea for the reference-point (RP) model comes from the reference point phenomenon which has been reported by behavioral and economic studies (De Martino et al., 2009; Baucells et al., 2011). According to this model, there is a distinct reference point for each context that is obtained by its expected rewards. Then the relative outcome of each option is calculated compared to this reference point. We implemented several forms of RP models considering the different forms of context reward (Palminteri et al., 2015). The RPD (Reference-Point Direct), RPA (Reference-Point Average), and RPM (Reference-Point Max) models, when the contextual rewards, *r*_*x*_, are considered to be direct *r*_*ch*_, an average of (*r*_*ch*_ + *Q*_*un*_)/2, and max(*r*_*ch*_, *Q*_*un*_), respectively, in the Partial version, and *r*_*ch*_, (*r*_*ch*_ + *r*_*un*_)/2, and max(*r*_*ch*_, *r*_*un*_) in the Complete version.


$$\delta_{x}=r_{x}-V_{x}$$

$$V_{x}=V_{x}+\alpha_{1}\delta_{x}$$

$$\delta_{ch}=\left(r_{ch}-V_{x}\right)-Q_{ch}$$

$$Q_{ch}=Q_{ch}+\alpha_{2}\delta_{ch}$$

where *V*_*x*_ is the value of the context, and *Q*_*ch*_ is the value of the chosen option. For the Complete version, we also update the unchosen options as below,


$$\delta_{un}=\left(r_{un}-V_{x}\right)-Q_{un}$$

$$Q_{un}=Q_{un}+\alpha_{3}\delta_{un}$$

In the Complete version, we used different versions for RP: one which only updates the chosen value, and one which updates both options with the same and different learning rates.

#### 4.3.3. The Difference (Dif) Model

In a context in which a participant is to maximize her rewards, the learning strategy is to find an advantageous option as soon as possible. The difference model is one of the models that allow fast detection of the advantageous option by learning the relative value. In this model, participants learn how much better the superior option is compared to the inferior option (Klein et al., 2017).


$$r_{rlt}=r_{FC}-r_{CF}$$

$$\delta =r_{rlt}-Q_{ch}$$

$$Q_{ch}=Q_{ch}+\alpha \delta$$

This model was only applied for the Complete version.

#### 4.3.4. The Hybrid (Hyb) Model

It has been shown that people are not fully absolute or fully relative learners. Rather they are hybrid learners who use both strategies but weight them differently (Bavard et al., 2018).


$$r_{abs}=r_{FC},\;r_{rlt}=r_{FC}-r_{CF}$$

$$r_{hyb}=wr_{abs}+\left(1-w\right)r_{rlt}$$

$$\delta =r_{hyb}-Q_{ch}$$

$$Q_{ch}=Q_{ch}+\alpha \delta$$

For the Partial version, we used the *Q*_*un*_ instead of *r*_*CF*_.

#### 4.3.5. The Forgetting Q-Learning (FQL) Model

In the Forgetting model, when the chosen value is updated by its prediction error, the unchosen value decays at a different learning rate (Barraclough et al., 2004; Ito and Doya, 2009; Katahira, 2015; Niv et al., 2015; Kato and Morita, 2016).


$$\delta_{ch}=r_{ch}-Q_{ch}$$

$$Q_{ch}=Q_{ch}+\alpha_{1}\delta_{ch}$$

$$Q_{un}=\alpha_{2}*Q_{un}$$

#### 4.3.6. The Experience-Weighted Attraction (EWA) Model

It has been shown that, in addition to counterfactual outcomes, the number of times an option is chosen has a substantial effect on value learning. Therefore, Camerer and Hua Ho (1999) brought these two features together in an augmented version of the Rescorla-Wagner model called the experience-weighted attraction model,


N(t+1)=ρN(t)+1

$$Q_{ch}=\left(Q_{ch}N\left(t\right)\varphi +r_{ch}\right)/N\left(t+1\right)$$

$$Q_{un}=\left(Q_{un}N\left(t\right)\varphi +\delta r_{un}\right)/N\left(t+1\right)$$

Where *N* is the *experience weight* of the option that is decayed with parameter ρ. The option value is also decayed with parameter φ. If there is a counterfactual outcome (similar to our Complete feedback version), the counterfactual outcome also affects the updating of the unchosen value with weight δ, but if there is not a counterfactual outcome (similar to our Partial feedback version), this parameter is zero.


N(t+1)=ρN(t)+1

$$Q_{ch}=\left(Q_{ch}N\left(t\right)\phi +r_{ch}\right)/N\left(t+1\right)$$

#### 4.3.7. The Sample-Based Episodic (SBE) Model

The idea of the sample-based episodic model is to calculate option values based on a recency-based sampling strategy rather than tracking the running average of option values (q-learning model; Bornstein and Norman, 2017; Bornstein et al., 2017). To estimate the value of option *a* at trial *t*, denoted by *Q*(*a*), this model stochastically samples one observed reward *r*_*i*_ with the following probability:


$$P\left(Q_{a}=r_{i}\right)=\alpha {\left(1-\alpha \right)}^{\left(t-i\right)}$$

By this probability, it is most (exponentially) likely to sample the most recent experience. Therefore, the likelihood, the probability of the behavioral data given this model, is computed as the following:


$$\sum_{j=1}^{t-1}\left[P(Q_{ch}=r_{j}).\;\sum_{k=1}^{t-1}\left[P(Q_{un}=r_{k}).\frac{e^{\beta Q_{ch}}}{e^{\beta Q_{ch}}+e^{\beta Q_{un}}}\right]\right]$$

This is a weighted sum of softmax probability over all possible pairs of competing options. In this model, any sample probability for trials with no rewards to sample from was set to 1.

#### 4.3.8. The Relative Asymmetric (RelAsym) Model

This RelAsym model consists of two relative value learning component (thorough reference point mechanism) and asymmetric updating component (thorough confirmation bias mechanism; Garcia et al., 2021; Ting et al., 2021). In the reference-point model, outcomes are context-dependent and causes that options' values to be learned relative to their reference-point. In the asymmetric updating of option-values, there is a tendency to update the values with positive prediction errors with a larger weight. The reference-point part of the model is as the following:


$$\delta_{x}=r_{x}-V_{x}$$

$$V_{x}=V_{x}+\alpha_{1}\delta_{x}$$

$$\delta_{ch}=\left(r_{ch}-V_{x}\right)-Q_{ch}$$

$$\delta_{un}=\left(r_{un}-V_{x}\right)-Q_{un}$$

where *V*_*x*_ is the value of the context. The confirmation part of the model is as the following:


$$\{\begin{array}{ccc} Q_{ch}=Q_{ch}+\alpha_{conf}\delta_{ch} & \text{if} & \delta_{ch}>0\\ Q_{ch}=Q_{ch}+\alpha_{disc}\delta_{ch} & \text{if} & \delta_{ch}<0\\ Q_{un}=Q_{un}+\alpha_{conf}\delta_{un} & \text{if} & \delta_{un}<0\\ Q_{un}=Q_{un}+\alpha_{disc}\delta_{un} & \text{if} & \delta_{un}>0 \end{array}$$

where *Q*_*ch*_ and *Q*_*un*_ are the values of the chosen option and unchosen option, and α_*conf*_ and α_*disc*_, are learning rates for confirmatory and disconfirmatory information.

#### 4.3.9. The Opposing Learning (OL) Model

The opposing learning model was inspired by the opposing role of dopamine on the chosen and unchosen options. In this model, both the chosen and unchosen values are simultaneously updated with the chosen prediction error, but in an opposite direction.


$$\delta_{ch}=r_{ch}-Q_{ch}$$

$$Q_{ch}=Q_{ch}+\alpha_{1}\delta_{ch}$$

$$Q_{un}=Q_{un}-\alpha_{2}\delta_{ch}$$

We extended this model for the Complete version by replacing the absolute reward with the weighted combination of absolute and relative rewards (a hybrid strategy).


$$r_{abs}=r_{FC},\;r_{rlt}=r_{FC}-r_{CF}$$

$$r_{hyb}=wr_{abs}+\left(1-w\right)r_{rlt}$$

$$\delta =r_{hyb}-Q_{ch}$$

$$Q_{ch}=Q_{ch}+\alpha_{1}\delta$$

$$Q_{un}=Q_{un}-\alpha_{2}\delta$$

### 4.4. Pure Simulation Procedure

The OL behavior has been examined in a wide range of task and parameter settings. Without loss of generality, we did the simulation with normalized settings such that we had σ = 1 in reward distributions. As an example, the normalized version of the setting of task N(μ = 64, σ = 10), parameters of β = 0.01, and any α_1_, α_2_, changes to its normalized version of N(μ = 6.4, σ = 1) (divide by 10), and parameters of β = 0.1 (multiply by 10), and the same α_1_, α_2_. The task settings included 10 different pairs of options in which their relative values were covered {1, 2, …, 10} ([μ_1_, μ_2_]∈{[10, 9], [10, 8], …, [10, 0]}, and σ = 1). The parameter settings covered a wide range of β: {0, 0.025, 0.05, 0.075, 0.1, 0.1025, …, 0.4}∪ {0.5, 0.6, …, 1}, α_1_: {0.1, 0.2, …, 1}, and α_2_/α_1_: {0, 0.5, 0.75, 0.875, 0.93, 0.96, 0.980.992, 0.996, 0.998, 0.999, 1}.

### 4.5. Fitting and Simulation Procedure

The data fitting was implemented using the *fmincon* function of Matlab software (the MathWorks Inc., Natick, MA). The fittings were done with several initial points to have a higher probability of finding the global optimum, rather than getting stuck on a local optimum. We calculated the exceedance probabilities (xp) and protected exceedance probabilities (pxp) for the model-comparison part (Stephan et al., 2009; Rigoux et al., 2014). Since the number of trials for each participant is different, we have fed the BIC to the BMS toolbox. To estimate parameters, we optimized maximum a posteriori (MAP) using weakly informative priors of β(1.2, 1.2) for each parameter. It is worth noting that the option values are on a scale of 0 to 100, so that the range of the β parameter will be on a scale of much <1, thus, the β(1.2, 1.2) would be a proper prior in the model fitting (Table 4). The simulation for each participant was done on its best-fitted parameters for 100 repetitions and then the representative behavior of this agent was obtained by averaging over its repetitions.

**Table 4:** The estimated parameters.

**Parameters**							
**Parameter**	**Constraint**	**SQL**	**RPA**	**Dif**	**Hyb**	**OL_1_**	**OL_2_**
**Partial**							
β	0 ≤ β < inf	0.07 ± 0.03	0.12 ± 0.08		0.06 ± 0.04	0.02 ± 0.02	0.03 ± 0.02
α_1_	0 ≤ α_1_ ≤ 1	0.25 ± 0.26	0.26 ± 0.27		0.37 ± 0.29	0.26 ± 0.2	0.32 ± 0.23
α_2_	0 < α_2_ ≤ α_1_		0.34 ± 0.3				0.21 ± 0.18
*w*	0 ≤ *w* ≤ 1				0.55 ± 0.37		
**Complete**							
β	0 ≤ β < inf	0.12 ± 0.09	0.37 ± 0.24	0.37 ± 0.23	0.2 ± 0.15	0.11 ± 0.12	0.1 ± 0.1
α_1_	0 ≤ α_1_ ≤ 1	0.14 ± 0.16	0.1 ± 0.12	0.09 ± 0.08	0.21 ± 0.15	0.22 ± 0.15	0.26 ± 0.14
α_2_	0 < α_2_ ≤ α_1_		0.11 ± 0.13				0.19 ± 0.16
α_3_	0 ≤ α_3_ ≤ 1		0.35 ± 0.3				
*w*	0 ≤ *w* ≤ 1				0.28 ± 0.23	0.28 ± 0.17	0.32 ± 0.19

*Mean±SD*.

## Data Availability Statement

The datasets presented in this study can be found in online repositories. The names of the repository/repositories and accession number(s) can be found at: https://osf.io/emgph/.

## Ethics Statement

The studies involving human participants were reviewed and approved by the Ethics Committee of the Institute for Research in Fundamental Sciences. The patients/participants provided their written informed consent to participate in this study.

## Author Contributions

ZB designed and performed the experiment, analyzed the data, and drafted the manuscript. MN supervised the research and critically revised the manuscript. A-HV contributed to the conception and critically revised the manuscript. All authors contributed to the article and approved the submitted version.

## Conflict of Interest

The authors declare that the research was conducted in the absence of any commercial or financial relationships that could be construed as a potential conflict of interest.

## Publisher's Note

All claims expressed in this article are solely those of the authors and do not necessarily represent those of their affiliated organizations, or those of the publisher, the editors and the reviewers. Any product that may be evaluated in this article, or claim that may be made by its manufacturer, is not guaranteed or endorsed by the publisher.
