# Supplementary material for: Implicit Counterfactual Effect in Partial Feedback Reinforcement Learning: Behavioral and Modeling Approach
Source: Front Neurosci. 2022 May 10;16:631347. doi: 10.3389/fnins.2022.631347 (PMC9127865; doi:10.3389/fnins.2022.631347)
Supplement: Supplementary file 1 [file Data_Sheet_1.pdf]

Supplementary Material

Binomial Test

Now we explain in detail the procedure carried out for the binomial test that we used in section *Contextual effect*. The binomial test is a test of the statistical significance of the deviations of the ratio of number of successes in n independent trials from an expected ratio. In our case, the null hypothesis is that the participants in the transfer phase choose uniformly random between A_2 and A_1 in the (A_1,A_2) combinations. Thus, the probability of choosing A_2 (or A_1) is 0.5. Under the null hypothesis, the test statistic should have binomial distribution. We used the two-sided binomial test. In binomial test to each subject we assigned a single binary variable indicating whether or not the subject preferred A_2 over A_1.

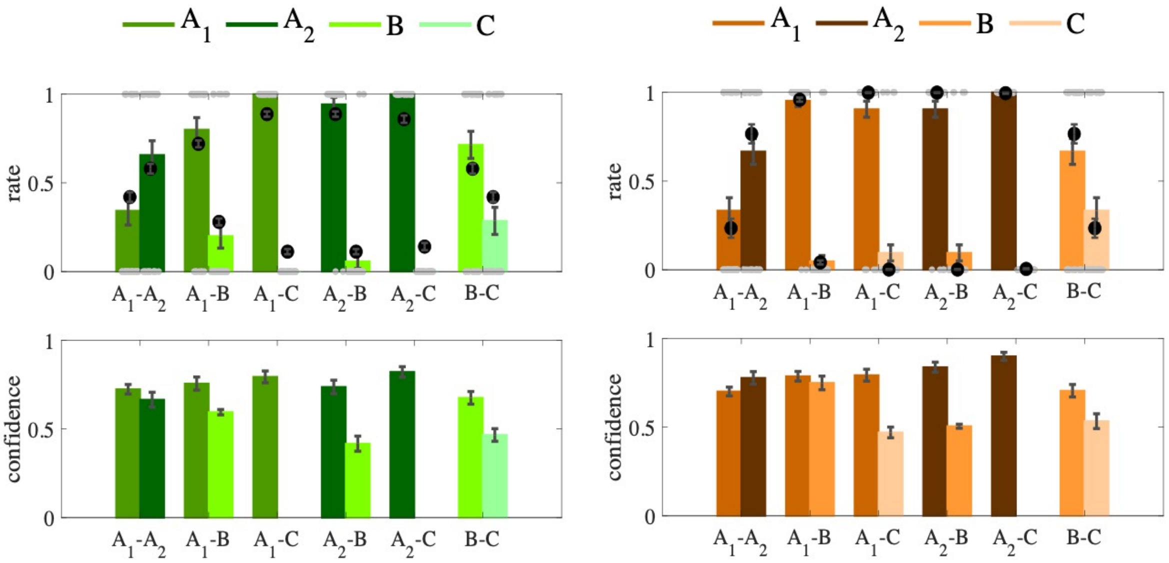

Figure S1. Behavioral results of the transfer phase. The participants' preferences in all 6 combinations (top), and corresponding confidences (bottom), with OL_1 predictions (black dots). The Partial feedback version is shown in green and the Complete feedback version in brown. Shadings denote *SD* and error bars denote *SEM*.

Name	Estimate	SE	tStat	DF	pValue	Lower	Upper
**Intercept**	0.05728	0.039288	1.458	15617	0.14487	-0.019729	0.13429
**vdif**	1.3182	0.09746	13.526	15617	$1.8916e-41$	1.1272	1.5092
**task2**	-0.067632	0.060265	-1.1222	15617	0.26178	-0.18576	0.050495
**vdif:task2**	2.0144	0.24154	8.3398	15617	$8.0551e-17$	1.541	2.4879

Table S1. Reward sensitivity in the Partial and Complete feedback versions. The hierarchical logistic regressions ($action \sim 1 + vdif * task + (1 + vdif * task|subject)$) were performed on the participants' choice behavior. The regressors are value difference between two competing options ($vdif$) with task version as a categorical variable (Partial is 1 and Complete is 2). The results illustrate that reward sensitivity was significantly higher in the Complete feedback version than in the Partial feedback version (pValue = $8.0551e-17$).

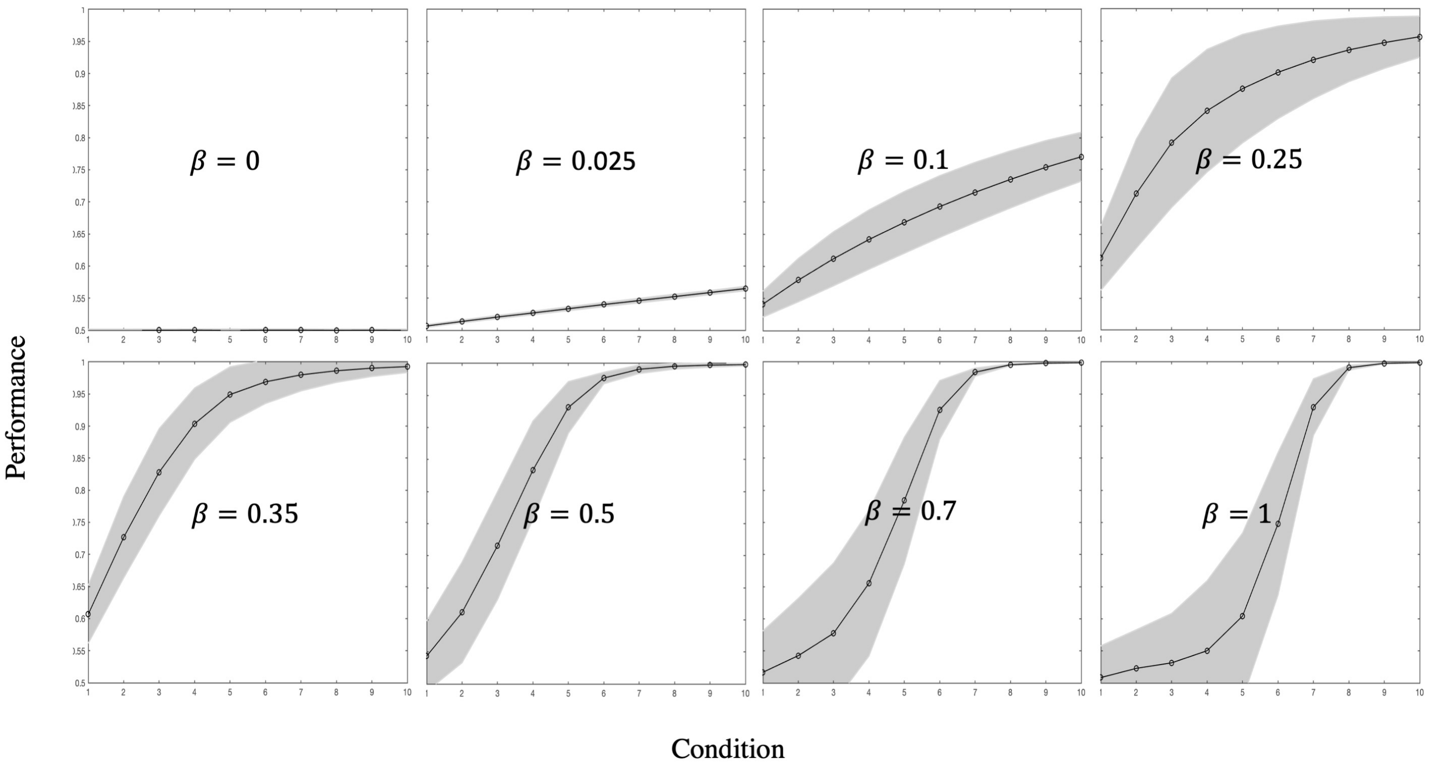

Figure S2. An OL agent's performance is better when the distance between option values is greater. The performance function changes with changing β. The task settings included 10 different pairs of options in which their relative values were covered $\{1, 2, ..., 10\}$ ($[\mu_1, \mu_2] \in \{[10, 9], [10, 8], ..., [10, 0]\}$, and $\delta = 1$). Performances were obtained by averaging over different α_1 and α_2.

Partial				
**parameter**	β	α_1	α_2	w
**constraint**	$0 \leq \beta < \inf$	$0 \leq \alpha_1 \leq 1$	$0 < \alpha_2 \leq \alpha_1$	$0 \leq w \leq 1$
**SQL**	0.07 ± 0.03	0.25 ± 0.26		
**FQL**	0.05 ± 0.02	0.43 ± 0.28	0.85 ± 0.16	
**RPD**	0.35 ± 0.31	0.18 ± 0.23	0.17 ± 0.28	
**RPA**	0.12 ± 0.08	0.26 ± 0.27	0.34 ± 0.3	
**RPM**	0.35 ± 0.31	0.18 ± 0.23	0.17 ± 0.28	
**EWA**	0.03 ± 0.02	0.56 ± 0.27	0.78 ± 0.24	
**Hyb**	0.06 ± 0.04	0.37 ± 0.29		0.55 ± 0.37
**OL$_1$**	0.02 ± 0.02	0.26 ± 0.2		
**OL$_2$**	0.03 ± 0.02	0.32 ± 0.23	0.21 ± 0.18	
**SBE**	0.07 ± 0.04	0.57 ± 0.24		
**RelAsym**	0.12 ± 0.08	0.29 ± 0.33	0.42 ± 0.29	0.24 ± 0.28

Complete					
**parameter**	β	α_1	α_2	α_3	w
**constraint**	$0 \leq \beta < \inf$	$0 \leq \alpha_1 \leq 1$	$0 < \alpha_2 \leq \alpha_1$	$0 \leq \alpha_3 \leq 1$	$0 \leq w \leq 1$
**SQL**	0.12 ± 0.09	0.14 ± 0.16			
**QL$_{21}$**	0.37 ± 0.23	0.09 ± 0.08			
**QL$_{22}$**	0.3 ± 0.2	0.11 ± 0.1	0.09 ± 0.08		
**FQL**	0.08 ± 0.05	0.24 ± 0.18	0.74 ± 0.27		
**RPA$_1$**	0.37 ± 0.23	0.09 ± 0.08	0.5 ± 0		
**RPA$_2$**	0.37 ± 0.24	0.1 ± 0.12	0.11 ± 0.13	0.35 ± 0.3	
**RPM$_1$**	0.37 ± 0.23	0.09 ± 0.08	0.5 ± 0		
**RPM$_2$**	0.36 ± 0.23	0.11 ± 0.13	0.11 ± 0.13	0.42 ± 0.34	
**EWA**	0.15 ± 0.18	0.74 ± 0.16	0.61 ± 0.25	0.8 ± 0.14	
**Dif**	0.37 ± 0.23	0.09 ± 0.08			
**Hyb**	0.2 ± 0.15	0.21 ± 0.15			0.28 ± 0.23
**OL$_1$**	0.11 ± 0.12	0.22 ± 0.15			0.28 ± 0.17
**OL$_2$**	0.1 ± 0.1	0.26 ± 0.14	0.19 ± 0.16		0.32 ± 0.19
**SBE**	0.52 ± 0.29	0.18 ± 0.15			
**RelAsym**	0.34 ± 0.21	0.27 ± 0.28	0.13 ± 0.17	0.28 ± 0.25	

Table S2. The estimated parameters. $Mean \pm SD$. The corresponding parameters for the Experience-Weighted Attraction model are β, ρ, and ϕ in the Partial feedback version, and β, δ, ρ, and ϕ in the Complete feedback version.

Partial	xp	pxp	*Complete*	xp	pxp
**SQL**	$1e-06$	$1.0001e-06$	**SQL**	0.001759	0.0017594
**RPD**	0	$6.4975e-11$	**QL_{21}**	0.24633	0.24633
**RPA**	0	$6.4975e-11$	**QL_{22}**	$5e-06$	$5.4329e-06$
**RPM**	0	$6.4975e-11$	**RPA_1**	$3e-06$	$3.4329e-06$
**Hyb**	0	$6.4975e-11$	**RPA_2**	$6e-06$	$6.4329e-06$
**OL_1**	1	1	**RPM_1**	$2e-06$	$2.4329e-06$
**OL_2**	0	$6.4975e-11$	**RPM_2**	$1.2e-05$	$1.2433e-05$
**FQL**	0	$6.4975e-11$	**Dif**	0.24653	0.24653
**EWA**	0	$6.4975e-11$	**Hyb**	0.001382	0.0013824
**SBE**	0	$6.4975e-11$	**OL_1**	0.5039	0.5039
**RelAsym**	0	$6.4975e-11$	**OL_2**	$5e-06$	$5.4329e-06$
			**FQL**	$5.6e-05$	$5.6433e-05$
			**EWA**	0	$4.3295e-07$
			**SBE**	$3e-06$	$3.4329e-06$
			**RelAsym**	$6e-06$	$6.4329e-06$

Table S3. Model comparison. Bayesian exceedance probability (xp), and protected exceedance probability of the learning phase.

Partial

all iterations	nll	BIC		
	learning	learning	learning + transfer(A_1A_2))	learning + transfer(all))
**SQL**	88.18 ± 5.49	186.82 ± 11.05	192.41 ± 11.04	205.38 ± 10.92
**RPD**	87.17 ± 5.49	190.04 ± 11.11	195.73 ± 11.12	208.91 ± 11.04
**RPA**	87.69 ± 5.47	191.07 ± 11.06	196.8 ± 11.09	209.56 ± 11
**RPM**	87.18 ± 5.49	190.05 ± 11.11	195.72 ± 11.11	208.91 ± 11.04
**Hyb**	86.68 ± 5.48	189.05 ± 11.07	194.87 ± 11.09	207.98 ± 10.99
**OL$_1$**	84.7 ± 5.49	179.86 ± 11.06	184.93 ± 11.05	198.09 ± 10.98
**OL$_2$**	83.66 ± 5.37	183.01 ± 10.86	188.33 ± 10.85	201.63 ± 10.75
**FQL**	83.05 ± 5.4	181.8 ± 10.9	187.03 ± 10.9	199.75 ± 10.81
**EWA**	87.25 ± 5.48	190.19 ± 11.08	195.79 ± 11.08	208.66 ± 10.98
**SBE**	107.29 ± 6.4	225.04 ± 12.91	232.23 ± 12.72	256.51 ± 12.97
**RelAsym**	86.92 ± 5.48	194.77 ± 11.12	199.9 ± 11.12	212.37 ± 11.15

Complete

	nll	BIC		
	learning	learning	learning + transfer(A_1A_2))	learning + transfer(all))
**SQL**	54.34 ± 4.98	119.24 ± 9.99	125.7 ± 10.01	145.2 ± 10.27
**QL$_{21}$**	51.71 ± 4.99	113.98 ± 10.01	123.4 ± 9.84	145.05 ± 10.69
**QL$_{22}$**	50.11 ± 5.01	116.05 ± 10.08	125.84 ± 9.87	148.06 ± 10.76
**RPA$_1$**	51.71 ± 4.99	119.25 ± 10.03	127.79 ± 9.72	145.57 ± 9.84
**RPA$_2$**	48.45 ± 4.99	118 ± 10.05	127.69 ± 9.69	146.72 ± 9.76
**RPM$_1$**	51.71 ± 4.99	119.25 ± 10.03	125.91 ± 9.86	144.8 ± 10.19
**RPM$_2$**	47.81 ± 5	116.73 ± 10.07	124.81 ± 9.78	147.99 ± 10.69
**Dif**	51.71 ± 4.99	113.98 ± 10.01	122.41 ± 9.68	139.98 ± 9.79
**Hyb**	48.94 ± 5	113.72 ± 10.05	122.19 ± 9.87	142.2 ± 9.85
**OL$_1$**	47.98 ± 5	111.79 ± 10.05	118.83 ± 9.88	136.68 ± 10.07
**OL$_2$**	47.53 ± 4.96	116.17 ± 9.99	123.25 ± 9.83	141.19 ± 9.91
**FQL**	50.41 ± 4.96	116.65 ± 9.96	123.24 ± 9.85	139.14 ± 9.91
**EWA**	47.34 ± 4.96	115.78 ± 9.99	124.85 ± 9.8	142.54 ± 10.11
**SBE**	78.47 ± 4.27	167.5 ± 8.62	201.11 ± 11.38	291.25 ± 18.5
**RelAsym**	48.96 ± 4.9	119.02 ± 9.86	128.24 ± 9.55	147.36 ± 9.84

Table S4. Model-comparison. Bayesian information criterion (BIC) of three different parts, learning phase, learning and (A_1A_2) of the transfer phase, and learning and all six combinations of the transfer phase for model space. nll indicates negative log likelihood. $Mean \pm SD$.
